# Synthesis and Characterization of an Epidermal Growth Factor Receptor‐Selective Ru^II^ Polypyridyl–Nanobody Conjugate as a Photosensitizer for Photodynamic Therapy

**DOI:** 10.1002/cbic.201900419

**Published:** 2019-10-22

**Authors:** Johannes Karges, Marta Jakubaszek, Cristina Mari, Kristof Zarschler, Bruno Goud, Holger Stephan, Gilles Gasser

**Affiliations:** ^1^ Chimie ParisTech PSL University CNRS Institute of Chemistry for Life and Health Sciences Laboratory for Inorganic Chemical Biology 75005 Paris France; ^2^ Institut Curie PSL University CNRS UMR 144 26 rue d'Ulm 75005 Paris France; ^3^ Department of Chemistry University of Zürich Winterthurerstrasse 190 8057 Zürich Switzerland; ^4^ Helmholtz-Zentrum Dresden–Rossendorf Institute of Radiopharmaceutical Cancer Research Bautzner Landstrasse 400 01328 Dresden Germany

**Keywords:** bioinorganic chemistry, medicinal inorganic chemistry, metal-based drugs, metals in medicine, photodynamic therapy

## Abstract

There is a current surge of interest in the development of novel photosensitizers (PSs) for photodynamic therapy (PDT), as those currently approved are not completely ideal. Among the tested compounds, we have previously investigated the use of Ru^II^ polypyridyl complexes with a [Ru(bipy)_2_(dppz)]^2+^ and [Ru(phen)_2_(dppz)]^2+^ scaffold (bipy=2,2′‐bipyridine; dppz=dipyrido[3,2‐a:2′,3′‐*c*]phenazine; phen=1,10‐phenanthroline). These complexes selectively target DNA. However, because DNA is ubiquitous, it would be of great interest to increase the selectivity of our PDT PSs by linking them to a targeting vector in view of targeted PDT. Herein, we present the synthesis, characterization, and in‐depth photophysical evaluation of a nanobody‐containing Ru^II^ polypyridyl conjugate selective for the epidermal growth factor receptor (EGFR) in view of targeted PDT. Using ICP‐MS and confocal microscopy, we could demonstrate that our conjugate has high selectivity for the EGFR receptor, which is a crucial oncological target because it is overexpressed and/or deregulated in a variety of solid tumors. However, in contrast to expectations, this conjugate was found to not produce reactive oxygen species (ROS) in cancer cells and is therefore not phototoxic.

## Graphical abstract

**Specificity comes to light**: Ru^II^ polypyridyl complexes used in photodynamic therapy (PDT) can target DNA. However, because DNA is ubiquitous, the selectivity of such photosensitizers could be increased by linking them to a targeting vector. Herein we present the synthesis, characterization, and in‐depth photophysical evaluation of a nanobody‐containing Ru^II^ polypyridyl conjugate selective for the epidermal growth factor receptor (EGFR) in view of targeted PDT.

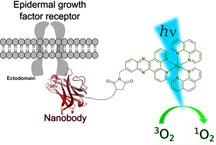

## Introduction

The use of photodynamic therapy (PDT) has expanded the possible techniques in medicine to treat various types of cancer (e.g., lung, bladder, esophageal and brain cancer) as well as bacterial, fungal or viral infections. Its effect is caused by a combination of an ideally nontoxic photosensitizer (PS), oxygen and light. Upon light exposure, the PS is able to produce reactive oxygen species (ROS), such as singlet oxygen (^1^O_2_) or other radicals. Due to the high reactivity of the latter, these can cause oxidative stress and damage in different cellular compartments (e.g., membrane, nucleus, endoplasmic reticulum, lysosome, mitochondria), leading ultimately to cell death.^1^

Next to the already approved PDT PSs, which are based on a tetrapyrrolic scaffold (i.e., porphyrins, chlorins, phthalocyanines), the development of Ru^II^ polypyridyl complexes as PDT PSs is receiving more attention due to their ideal photophysical and photochemical properties, which include, among others, high water solubility, high chemical stability and photostability, intense luminescence, large Stokes shifts, high ^1^O_2_ production.1a–1d, 2 These attractive features have allowed one of such complexes, namely TLD‐1433, to enter into clinical trial as a PDT PS against bladder cancer.^3^ Phase II has been recently started.2f

**Figure 1:**
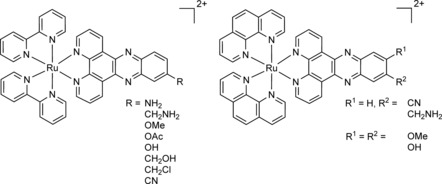
Structures of [Ru(bipy)_2_(dppz)]^2+^ and [Ru(phen)_2_(dppz)]^2+^ complexes as PSs developed by our group.1a, 2c, 4

In this context, our group was able to demonstrate that Ru^II^ complexes of the type [Ru(bipy)_2_(dppz)]^2+^ (bipy=2,2′‐bipyridine, dppz=dipyrido[3,2‐a:2′,3′‐*c*]phenazine) and [Ru(phen)_2_(dppz)]^2+^ (phen=1,10‐phenanthroline) were effective PDT PSs (Figure 1).1a, 2c, 4 As a highlight, we could demonstrate that some of these complexes were nontoxic in the dark and highly toxic upon light irradiation with IC_50_ values in the low micromolar range and a phototoxic index of up to >150.2c Based on the extended planar π‐system of the dppz ligand, which is able to intercalate into the base pairs of the DNA, these compounds showed a preferable nuclear localization. Upon light exposure, these complexes caused oxidative stress, as well as DNA photocleavage, suggesting that they impaired replication and integrity of the genetic material.1a, 2c, 4

Highly proliferating cells like cancer cells are generally preferably targeted by such compounds over healthy cells, as it is the case for cisplatin.^5^ However, other frequently dividing cells in the organism (e.g., hair follicles, gastrointestinal tract, bone marrow) can be affected, leading to severe side effects for the patients.4a, 6 Thus, it is extremely important to increase the selectivity of PDT PS, for example, with the development of a suitable delivery system.

So far, the examples of Ru^II^ polypyridyl complexes for targeted PDT are scare, if we do not take into account polymer encapsulation/nanoparticle attachment.4a, 7 The group of Lilge could recently demonstrate that the premixing of TLD‐1433 with transferrin was able to increase the extinction coefficient, prolongs the absorption range, reduced photobleaching, cellular uptake as well as overall toxicity of the compound.^8^ Our group previously demonstrated the efficiency of the coupling of a metal‐based PDT PS to peptides, which are known to bind specifically to abundant molecular targets on malignant cells. More precisely, in those studies, bombesin, that is known to target the human gastrin‐releasing peptide receptor as well as a nuclear localization signal peptide that facilitates the intracellular transport into the nucleus were coupled to Ru‐based PDT PSs. We were able to demonstrate an increased uptake of the conjugate in the receptor‐expressing cells in comparison with the free complex.4a The groups of Weil and Rau were able to link the peptide hormone somatostatin to a PS and could show a 100‐fold increased efficiency for somatostatin receptor‐expressing cells relative to the free PS.7a Recently, the same authors described a macromolecular plasma protein serum albumin‐PS conjugate with several Ru complexes bound to the protein surface. Using the protein as a nanocarrier, the PSs were delivered selectively to the mitochondria, where it showed an impressive phototoxicity with IC_50_ values in the nanomolar range.7c Notably, a variety metal complexes as for example Re^I^, Pt^II^, Ru^II^ or Ir^III^ compounds have been successfully coupled to peptides to increase receptor selectivity.^9^

Among the different established classes of delivery systems^10^ (e.g., oil dispersions, encapsulation in polymeric particles/lysosomes, targeting peptide‐PS conjugates, polymer–PS conjugates), the conjugation of PS to monoclonal antibodies (mAb) takes advantage of the excellent target specificity of the latter. However, despite their clinical success, the concept of using mAb‐PS conjugates is afflicted with several important drawbacks. These vector molecules are known for their high stability and prolonged serum half‐life, slow pharmacokinetics and clearance from the body. This leads to an increase of the absolute level of the mAb‐PS conjugate in the tumor alongside with an increased nonspecific uptake in non‐target tissues.^11^ Additionally, the treatment of solid tumors is limited due to penetration problems of the large conjugate into the tumor caused by poor vascularization, drainage, interstitial pressure and dense stroma.^12^ An attractive strategy to circumvent these limitations is the use of smaller oncotropic vector molecules like antibody fragments or nanobodies (NBs).^13^ NBs represent the antigen‐binding domain of heavy‐chain‐only antibodies that occur in species belonging to the family of *Camilidae*. Their small size, stability, solubility, fast pharmacokinetics as well as high specificity and affinity for their cognate antigens make them powerful targeting agents for diagnostic imaging and targeted therapy.^14^ Notably in this context, Caplacizumab, a bivalent anti‐von Willebrand factor NB, is currently in phase III clinical trials against acquired thrombotic thrombocytopenic purpura.^15^

A recent study has highlighted the high tumor uptake, rapid blood clearance and low liver uptake of a ^99m^Tc‐labeled NB as an imaging probe for epidermal growth factor receptor (EGFR) positive tumors.^16^ This receptor, which is involved in many cellular processes such as proliferation, differentiation and cell survival, represents a crucial target in oncology as it is overexpressed and/or deregulated in a variety of solid tumors, including head and neck, breast, non‐small‐cell lung and pancreatic cancer. Therefore, EGFR is a major target for cancer therapy.^16^, ^17^ Notably, the successful conjugation of the PS IRDye700DX‐maleimide to nanobodies for hepatocyte growth factor receptor targeted PDT was recently demonstrated.^18^

With this in mind, we report herein the design, synthesis, characterization and in‐depth biological evaluation of a NB‐containing Ru^II^ polypyridyl conjugate. The conjugate consists of three building blocks: 1) A [Ru(phen)_2_(dppz)]^2+^ complex, which is known to have an excellent phototoxicity,1a, 2c, 4 2) a 7C12 NB, which is known for specific binding to EGFR expressing cells^16^, ^19^ and 3) a peptide chain with a poly‐glycine unit, which is necessary for an efficient and site‐specific conjugation by a sortase A (SrtA)‐mediated *trans*‐peptidation reaction leading to an 1:1 NB:PS ratio.^20^ To the best of our knowledge, we report herein the first NB‐containing Ru^II^ polypyridyl conjugate as a PDT PS for EGFR‐targeted PDT. As can be seen below, thanks to this design, a highly selective NB‐containing [Ru(phen)_2_(dppz)]^2+^ conjugate Ru‐NB could be unveiled.

## Results and Discussion

### Synthesis of the [Ru(phen)_2_(dppz‐7‐maleimidemethyl‐S‐Cys‐(Ser)_2_(Gly)_5_‐NH_3_)] complex

**Scheme 1:**
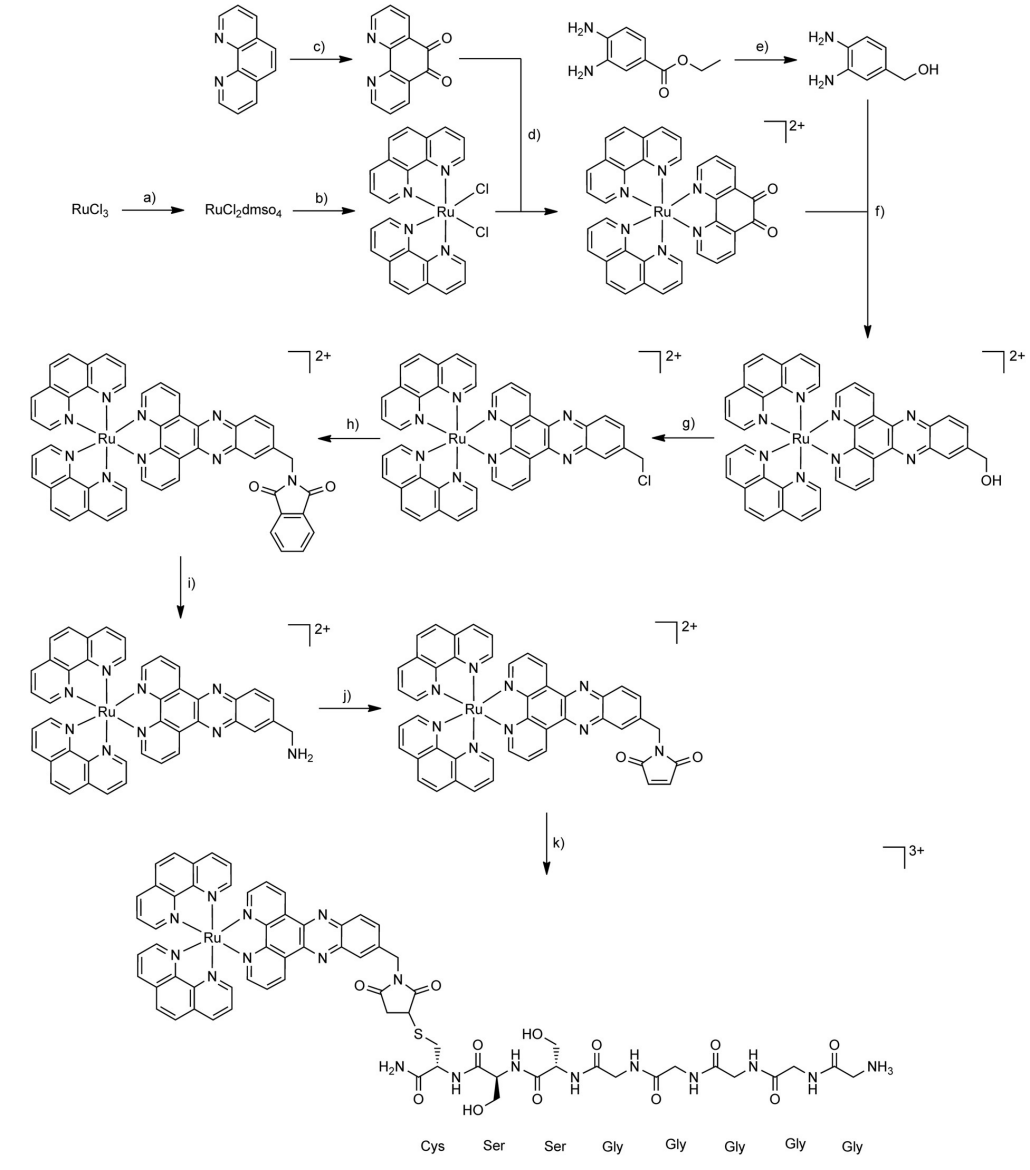
Total synthesis of [Ru(phen)_2_(dppz‐7‐maleimidemethyl‐S‐Cys‐(Ser)_2_(Gly)_5_‐NH_3_)](TFA)_3_. a) EtOH, reflux 3 h, DMSO, 150 °C 2 h; b) 1,10‐phenanthroline, LiCl, DMF, reflux overnight under N_2_; c) 1,10‐phenanthroline, KBr, H_2_SO_4_, HNO_3_, 90 °C 3 h under N_2_; d) EtOH, 80 °C 3 h under N_2_; e) LiAlH_4_, THF, 60 °C 1 h under N_2_; f) acetic acid, CH_3_CN, reflux 1 h under N_2_; g) (COCl)_2_, DMF, CH_3_CN, RT, overnight under N_2_; h) phthalimide, K_2_CO_3_, DMF, RT, overnight; i) NH_2_NH_2_, MeOH, reflux overnight under N_2_; j) maleic anhydride, AcOH, reflux 10 h under N_2_; k) (NH_3_‐(Gly)_5_‐(Ser)_2_‐Cys‐CONH_2_)(TFA), CH_3_CN:H_2_O 1:1, RT, 30 h.

The synthetic strategy for the synthesis of the [Ru(phen)_2_(dppz‐7‐maleimidemethyl‐S‐Cys‐(Ser)_2_(Gly)_5_‐NH_3_)]^3+^ complex is described in Scheme 1. The [Ru(phen)_2_(dppz‐7‐aminomethyl)](PF_6_)_2_ complex was synthesized as previously reported in nine synthetic steps.4a The synthesis of the [Ru(phen)_2_(dppz‐7‐maleimidemethyl)](PF_6_)_2_ complex is already published but, in this study, a slightly different experimental procedure was employed.4a The maleimide‐containing Ru^II^ complex was prepared by reacting the [Ru(phen)_2_(dppz‐7‐aminomethyl)](PF_6_)_2_ complex with maleic anhydride. [Ru(phen)_2_(dppz‐7‐maleimidemethyl)](PF_6_)_2_ was coupled to the poly‐glycine chain via a thio‐Michael addition reaction. As recently highlighted, this bioconjugation presents important advantages such as synthetic accessibility, excellent reactivity and, importantly, biocompatibility.^21^ Following this synthetic strategy, the thiosuccinimide product [Ru(phen)_2_(dppz‐7‐maleimidemethyl‐S‐Cys‐(Ser)_2_‐(Gly)_5_‐NH_3_)]^3+^ was prepared by treating the thiol of the (NH_3_‐(Gly)_5_‐(Ser)_2_‐Cys‐CONH_2_)(TFA) peptide chain with the [Ru(phen)_2_(dppz‐7‐maleimidemethyl)](PF_6_)_2_ complex. The product was obtained after an overnight reaction at room temperature and isolated via preparative HPLC. The identity of the obtained complexes was confirmed by HRMS and the purity verified by HPLC (Figures S1 and S2 in the Supporting Information). We note that S‐maleimide adducts^22^ have been found to have some problems of stability and this is the reason why alternative conjugation techniques are currently sought.

### Sortase A‐mediated conjugation

**Scheme 2:**
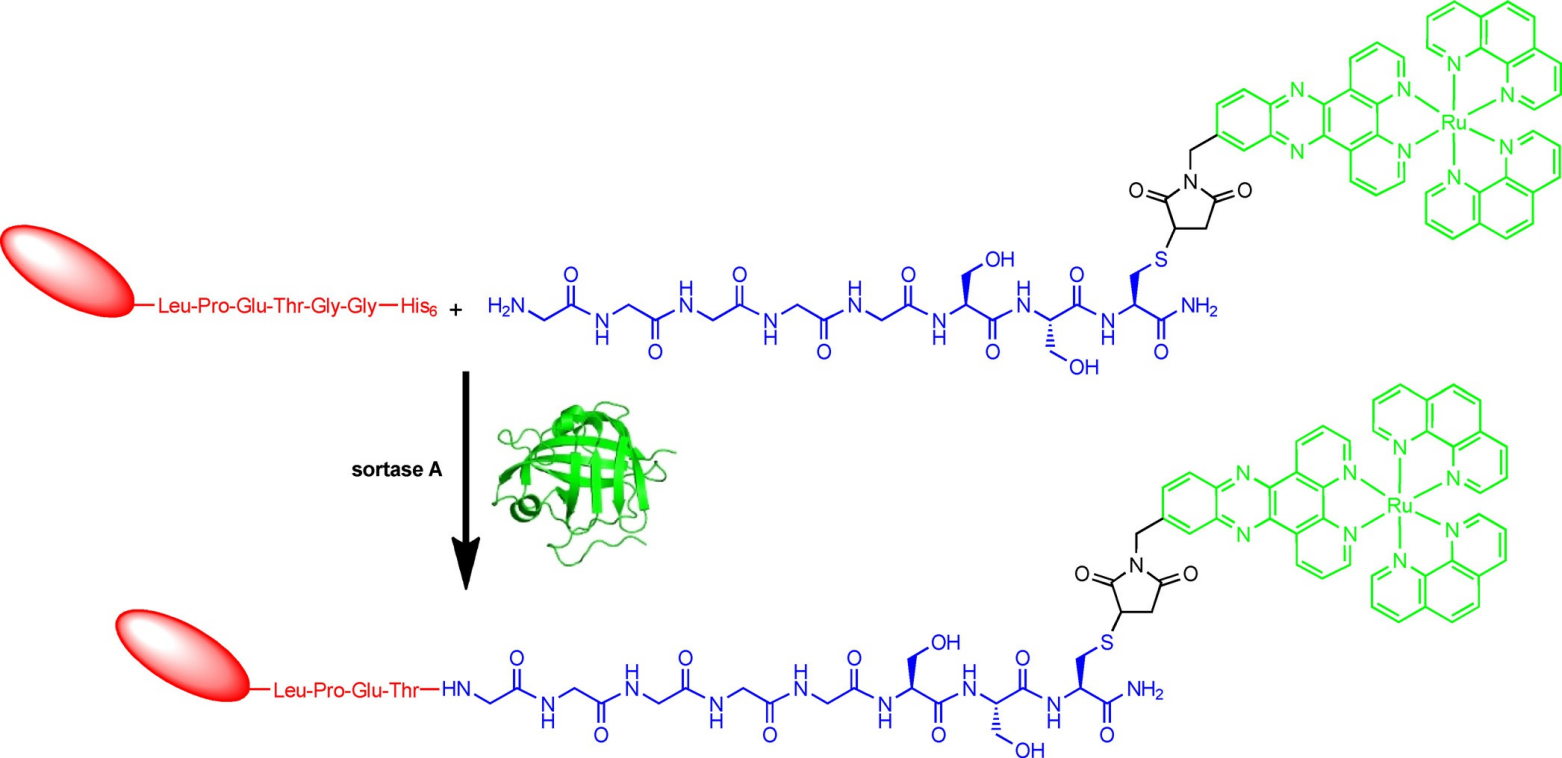
Overview of the sortase A‐mediated site‐specific modification of the NB derivative 7C12‐Strep‐Sortag‐His_6_ with the Ru(phen)_2_(dppz‐7‐maleimidemethyl‐*S*‐Cys‐(Ser)_2_(Gly)_5_‐NH_2_) complex resulting in Ru‐NB conjugate. The [Ru(phen)_2_(dppz)]^2+^ complex is highlighted in green, while the engineered NB is drawn in red and the peptide chain with a polyglycine unit is depicted in blue. PDB ID of sortase A from *Staphylococcus aureus*: https://www.rcsb.org/structure/1T2P.^23^

Site‐specific attachment of the [Ru(phen)_2_(dppz‐7‐maleimidemethyl‐S‐Cys‐(Ser)_2_‐(Gly)_5_‐NH_3_)]^3+^ complex to the EGFR‐specific NB 7C12 by sortase A requires protein engineering to endow the desired conjugation site at the C‐terminal end of the NB with the unique sortase recognition motif. To this end, the NB was produced with its C terminus tagged with a (GGGGS)_3_ spacer followed by a Strep‐tag, the LPETGG sortase motif, another (GGGGS)_3_ spacer and a hexahistidine purification tag (His_6_). As successful sortase A‐mediated conjugation leads to the elimination of the His_6_ tag, this design allows the removal of the unreacted NB as well as of the His_6_‐tagged enzyme by affinity chromatography (Scheme 2).

**Figure 2:**
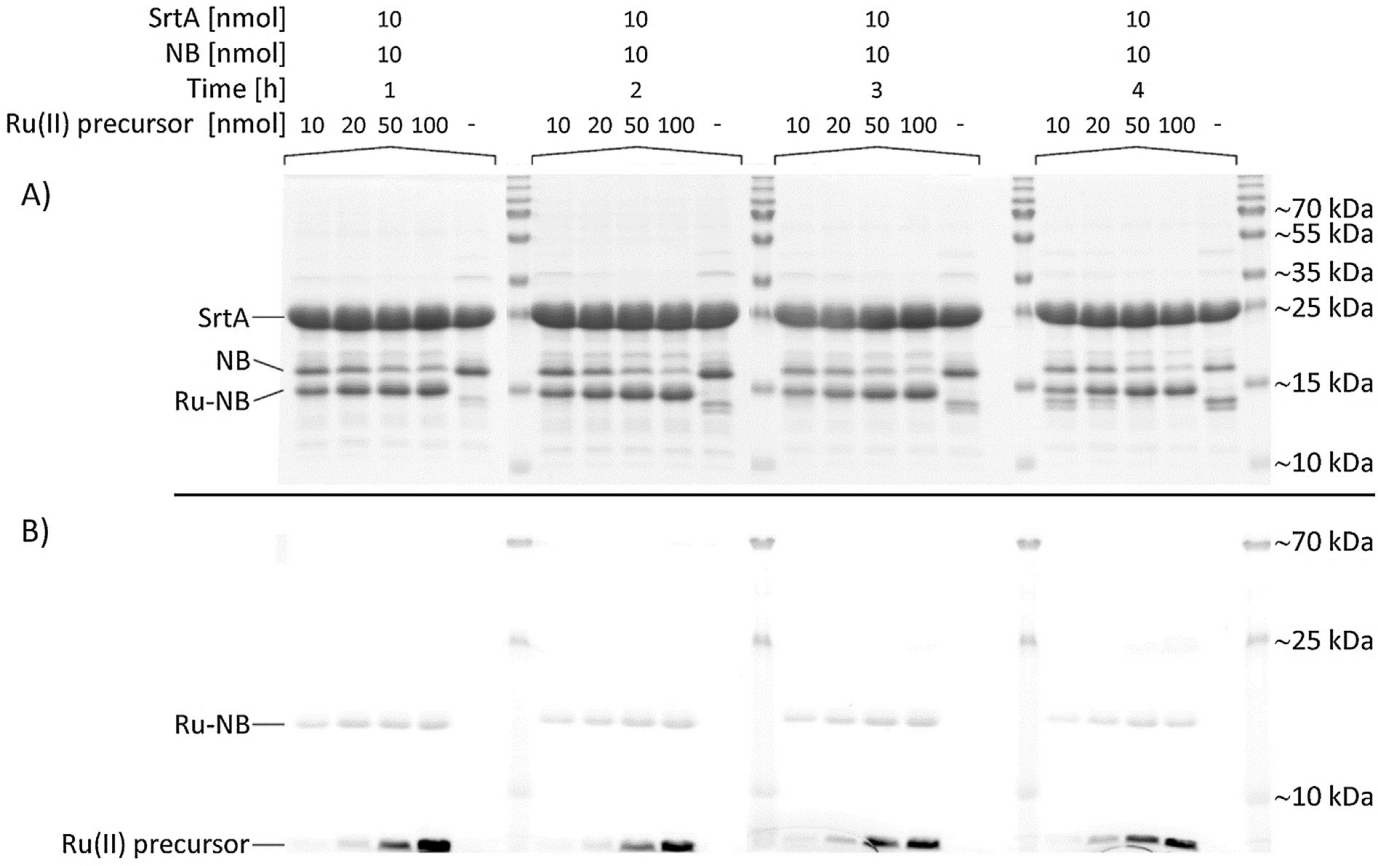
Course of reaction for the chemoenzymatic conjugation of [Ru(phen)_2_(dppz‐7‐maleimidemethyl‐S‐Cys‐(Ser)_2_(Gly)_5_‐NH_3_)]^3+^ to the EGFR‐specific NB 7C12. While the molar ratio between SrtA and NB was kept constant (1:1), the amount of the Ru^II^ precursor was increased (10–100 nmol) to finally achieve molar ratios of 1:1:1, 1:1:2, 1:1:5 and 1:1:10, respectively. The reaction was monitored for up to 4 h and aliquots were separated on 15 % SDS polyacrylamide gels. After electrophoresis, gels were imaged with a D‐DiGit Gel Scanner (B) to detect the signal of the Ru^II^ complex and subsequently stained with colloidal Coomassie G‐250 (A).

To optimize the sortase‐mediated bioconjugation reaction, the molar ratios of SrtA, NB and [Ru(phen)_2_(dppz‐7‐maleimidemethyl‐S‐Cys‐(Ser)_2_(Gly)_5_‐NH_3_)]^3+^ as well as the reaction time were varied (Figures S3 and S4). A 4 h reaction at 30 °C with a molar ratio of 1:1:10 was identified as being ideal (Figure 2).

Consequently, these conditions were kept in an upscaled reaction using 2 μmol SrtA, 2 μmol sdAb and 20 μmol [Ru(phen)_2_(dppz‐7‐maleimidemethyl‐S‐Cys‐(Ser)_2_(Gly)_5_‐NH_3_)]^3+^. After purification of the reaction mixture by affinity chromatography, the obtained conjugate 7C12‐Strep‐[Ru(phen)_2_(dppz‐7‐maleimidemethyl‐S‐Cys‐(Ser)_2_(Gly)_5_‐NH_3_)]^3+^ (Ru‐NB) was analyzed by MALDI‐TOF MS (Figure S5). The mass spectra of the final purified product Ru‐NB showed a homogeneous population of a single‐conjugated NB with a molecular mass of ≈17.7 kDa.

### Photophysical properties

With the conjugate in hand, we performed photophysical measurements to evaluate its potential as a PDT agent. At first, the absorptions of [Ru(phen)_2_(dppz‐7‐maleimidemethyl)](PF_6_)_2,_ [Ru(phen)_2_(dppz‐7‐maleimidemethyl‐S‐Cys‐(Ser)_2_(Gly)_5_‐NH_3_)](TFA)_3_ and Ru‐NB were measured to investigate if the peptide chain or the NB conjugation had an influence on the photophysical properties of the Ru^II^ polypyridyl complexes. Because the conjugate is insoluble in CH_3_CN, the measurements of Ru‐NB were performed in DMSO. The comparison between the absorption spectra (Figure S6) shows small differences that can be explained by solvent effects. As all major bands are still comparable, we assume that the conjugation did not change the photophysical properties of the Ru^II^ polypyridyl complex. As a second experiment, the luminescence of the conjugate was investigated upon excitation at 450 nm in DMSO. The maximum of the emission of the complex (Figure S7) was determined to be at 633 nm. Consequently, there is a large Stokes shift which results in minimal interference between excitation and luminescence. The luminescence quantum yield (*Φ*_em_) was measured upon excitation at 450 nm by comparison with the model complex [Ru(bipy)_3_]Cl_2_ in CH_3_CN (*Φ*_em_=5.9 %).^24^ The luminescence quantum yield (*Φ*_em_) of the conjugate Ru‐NB with a value of 3.3 % was found to be in the same range as other complexes of the type [Ru(bipy)_2_(dppz)]^2+^ and [Ru(phen)_2_(dppz)]^2+^.2c, 4 For a deeper investigation of the excited state, the luminescence lifetimes were determined in degassed and air saturated DMSO upon excitation at 450 nm to investigate the influence of the presence of oxygen. As expected, the luminescence lifetime in a degassed solution was much longer (589 ns, Figure S8) than in an aerated solution (134 ns, Figure S9). This shows that oxygen has a significant influence on the lifetime of the excited state and indicates that ^3^O_2_ can interact with the triplet state of the complex.

### Singlet oxygen generation

Knowing that the triplet excited state of the conjugate is able to interact with oxygen, we were interested in determining the singlet oxygen quantum yield *Φ*(^1^O_2_) of Ru‐NB using two methods previously described by our group,^25^ namely: 1) Direct by measurement of the phosphorescence of ^1^O_2_ at 1270 nm. Notably, this method is dependent on the used setup. With the used equipment in our laboratory, we can only detect *Φ*(^1^O_2_)>0.20; 2) Indirect by measurement of the change in absorbance of a reporter molecule which is monitored by UV/VIS spectroscopy. Because the measurements were performed in DMSO and aqueous solution, only rather small values (Table 1) could be measured. This is not surprising and has already been investigated for several other [Ru(bipy)_2_(dppz)]^2+^ and [Ru(phen)_2_(dppz)]^2+^ derivatives.2c, 4a, 4b In‐depth investigations showed that the excited state of the complex is quenched in an aqueous solution due to hydrogen bonding interactions between the nitrogen atoms of the dppz ligand and the solvent.^26^ Comparison of the singlet oxygen quantum yield of Ru‐NB with those obtained for structurally related [Ru(bipy)_2_(dppz)]^2+^ complexes,2c revealed that these values are in the same range. This strongly suggests that the bioconjugation did not significantly influence this property.

**Table 1:** Singlet oxygen quantum yields (*Φ*(^1^O_2_)) of Ru‐NB in DMSO and aqueous solution determined by direct and indirect method by excitation at 450 nm.^[a]^

DMSO direct	D_2_O direct	DMSO indirect	PBS indirect
n.d.	n.d.	9 %	4 %

[a] Average of three independent measurements, ±10 % (n.d.=not detectable).

### In vitro evaluation of EGFR targeting after conjugation

**Figure 3:**
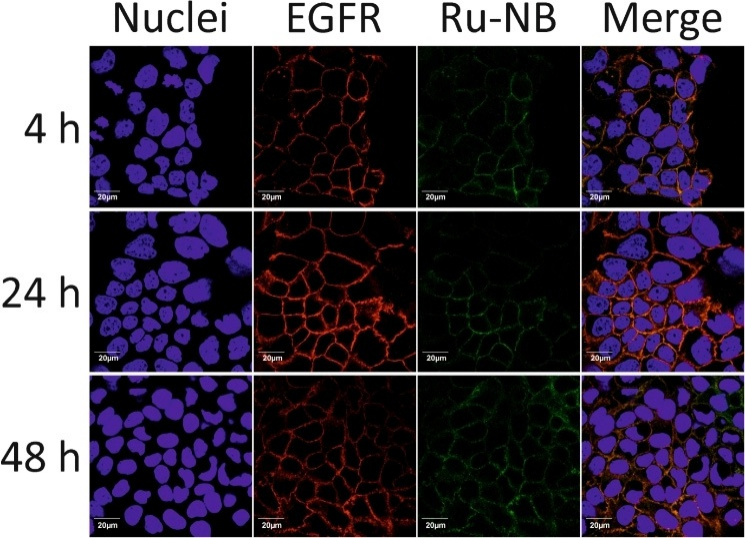
Confocal immunofluorescence microscopy images of A431 cells exposed to Ru‐NB for 4, 24, and 48 h at 37 °C showing specific binding and co‐localization of the single‐conjugated NB with EGFR. Scale bars: 20 μm.

To investigate the targeting ability of the functionalized NB, uptake in the human epithelial cell line A431 originating from an epidermoid carcinoma of the skin was examined by confocal fluorescence microscopy. These squamous carcinoma cells express approximately 2×10^6^ EGFR molecules per cell,^27^ which represents a high expression level. Confocal imaging of A431 cells showed co‐localization of Ru‐NB with EGFR (Figure 3), thus indicating the preserved targeting ability of 7C12 after site‐specific modification. Notably, Ru‐NB showed a predominant membrane staining even after 48 h of incubation at 37 °C, and only very little intracellular fluorescence was observed. However, it has been shown recently that the free amine ruthenium complex is characterized by a poor cellular uptake even at high micromolar concentrations.4a

### Cellular uptake of the bioconjugates

**Figure 4:**
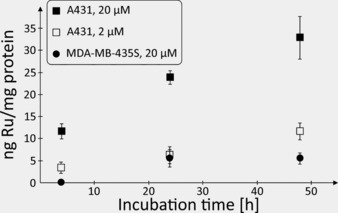
Amount of cell‐associated ruthenium after incubation of EGFR‐positive A431 and EGFR‐negative MDA‐MB‐435S cells with 2 or 20 μm of Ru‐NB for up to 48 h at 37 °C. The level of ruthenium in cell lysates of MDA‐MB‐435S exposed to 2 μm of Ru‐NB were below the analytical limit and are thus not shown.

The presence of a metal ubiquitous in a cellular environment as an essential component of the PS allows investigating the cellular accumulation of the bioconjugate by inductively coupled plasma–mass spectrometry (ICP‐MS).^28^ To demonstrate the receptor‐specific uptake, EGFR‐positive (A431) and EGFR‐negative (MDA‐MB‐435S) cells were incubated for different periods of time (4, 24, and 48 h) with various concentrations of the bioconjugate in the dark at 37 °C. The amount of cell‐associated ruthenium was determined by ICP‐MS and related to the cellular protein content (Figure 4). Although ruthenium was detectable in the cell lysate of both cell lines after 24 and 48 h, respectively, the amount of the metal strongly correlated with the level of EGFR expression. There was more of ruthenium in the EGFR‐overexpressing cell line than in the EGFR‐negative one. This finding confirmed that cell association was primarily mediated by the NB and not by the PS.

An identical cell uptake study was performed with the complex [Ru(bipy)_2_(dppz‐OMe)](PF_6_)_2_,2c resulting in similar ruthenium levels for the A431 cell line (Figure S10 and Table 2). The amount of ruthenium detected in MDA‐MB‐435S cells upon incubation with this non‐targeted Ru complex was higher at each time point relative to the EGFR‐targeting Ru‐NB conjugate. This result is unsurprising, as the latter cells lack these receptor proteins at their surface.

**Table 2:** Head‐to‐head comparison of uptake of Ru‐NB and [Ru(bipy)_2_(DPPZ‐OMe)](PF_6_)_2_ into A431 and MDA‐MB 435S cells.^[a]^

*t* [h]	Ru‐NB				Ru(bipy)_2_(DPPZ‐OMe) (PF_6_)_2_			
	A431		MDA‐MB 453S		A431		MDA‐MB 453S	
	Concentration of substance [μm]							
	2	20	2	20	2	20	2	20
4	3.26±1.30	11.67±1.70	<LOD	<LOD	1.20±0.28	8.54±2.23	1.18±0.14	5.20±0.90
24	6.20±1.86	23.84±1.54	<LOD	5.52±2.00	2.51±0.19	18.83±2.84	1.84±0.05	15.84±2.69
48	11.54±1.89	32.87±4.87	<LOD	5.45±1.32	5.75±0.74	46.94±1.89	1.92±0.08	19.93±2.39

[a] The amount of cell‐associated ruthenium [ng mg^−1^ protein] was measured by ICP‐MS.

To confirm the receptor specificity of the ruthenium accumulation, A431 cells were incubated in the presence or absence of cetuximab in addition to Ru‐NB. The epitope for 7C12 partially overlaps the cetuximab epitope on domain III of the EGFR extracellular region and an excess of the mAb can block its interaction with the receptor.^16^, ^29^ After 24 and 48 h of incubation with 200 nm of Ru‐NB at 37 °C, 0.77 and 2.74 ng ruthenium per mg protein (Table 3), respectively, were detected in the cell lysates. Upon co‐incubation of EGFR‐overexpressing A431 cells with Ru‐NB and cetuximab, no cell‐associated ruthenium was detectable even after 48 h.

**Table 3:** Amount of cell‐associated ruthenium after incubation of EGFR‐positive A431 with 200 nm of Ru‐NB for 24 or 48 h at 37 °C.^[a]^

	ng Ru per mg protein	
cetuximab	–	1 μm
24 h	0.77±0.10	<LOD
48 h	2.74±0.12	<LOD

[a] The level of ruthenium in cell lysates of A431 co‐incubated with 1 μm of the EGFR‐blocking antibody cetuximab were below the limit of detection (LOD).

These latter findings corroborate the hypothesis that cellular ruthenium association occurs in a receptor‐mediated manner. Overall, Ru‐NB targets EGFR specifically. Importantly, the free water‐soluble PS exhibits only poor cell binding capacity and lacks cell line selectivity, until their conjugation to targeting moieties. These facts together strongly provide the basis for tumor‐specific PDT.

### Dark cytotoxicity and phototoxicity of Ru‐NB

To evaluate the potency of the bioconjugate Ru‐NB as a PDT agent, its cytotoxicity in the dark and upon light irradiation was determined. For these experiments, the A431 cell line had to be chosen due to the strong light sensitivity of the MDA‐MB‐435S (EGFR negative) cell line that precluded it from phototoxicity studies. To avoid light sensitivity in A431 cell line, irradiation at 480 nm was performed in sequences. 6×3.5 min of irradiation with 15 min gap in between (6.741 J cm^−2^) were used. Dark treatment and surprisingly light irradiation of the A431 cells (48 h incubation with Ru‐NB) at 480 nm did not cause any cytotoxic effect (IC_50 dark_ >25 μm, IC_50 light_ >25 μm, see Figure S11) for Ru‐NB. We note that we could not go for higher concentration due to conjugate precipitation at 50 μm. Adding polyethylene glycol spacers, changing the ionic strength or the pH could possibly affect the conjugate solubility, and consequently help solving this problem. Lack of cytotoxicity encouraged us to try to enhance the internalization of the conjugate into the cells. For that purpose, an additional step was used, namely temperature change.^30^ Cells treated with Ru‐NB were incubated for 1 h at 4 °C. As EGFR internalization is an energy‐dependent process, incubating cells at 4 °C inhibits the endocytosis processes but not the binding of Ru‐NB to the receptor. A temperature shift to 37 °C (for 1 h) allowed then for efficient endocytosis of the receptor with the bound conjugate. This step enables a higher accumulation of Ru‐NB in the cells. Due to conjugate precipitation, the highest concentration tested was 35 μm. Ru‐NB was again found to be nontoxic in the dark (IC_50_ >35 μm). Unfortunately, light irradiation at 480 nm (6×3.5 min with 15 min gap between irradiations) again did not cause any phototoxic effect (IC_50_ >35 μm, see Figure S12).

### Cellular ROS production by Ru‐NB

The lack of phototoxicity of Ru‐NB led us to investigate whether this conjugate could produce ROS in irradiated cells. For that purpose, we have stained A431 cells with the known ROS probe DCFH‐DA (2′,7′‐dichlorodihydrofluorescein diacetate). Cells were then treated with Ru‐NB (35 μm) using the receptor internalization protocol, irradiated (480 nm light for 3.5 min; 1,124 J cm^−2^) and suspended in PBS buffer. The DCFH‐DA signal was detected using flow cytometry instrument. As can be seen in Figure S13, there was no ROS production in the A431 cells that were treated with Ru‐NB and then irradiated, as distinct from the H_2_O_2_ treated control. This unexpected result might be caused by the impairment of the internalization of Ru‐NB into the cells. Another explanation would be that the ROS produced are directly reacting with the NB itself. However, this hypothesis is unlikely, as ^1^O_2_ was detected during the ^1^O_2_ production measurements.

## Conclusion

In summary, in this article, we present the synthesis, characterization as well as photophysical and biological evaluation of a novel nanobody containing Ru^II^ polypyridine conjugate. As a benefit of the linkage to a 7C12 nanobody, the conjugate selectively accumulated at the epidermal growth factor receptor (EGFR). The investigation of the uptake via ICP‐MS indicated that the conjugate has been successfully internalized inside cancerous A431 cells. Photophysical studies in cuvette suggested that the photophysical properties of the conjugate remain unchanged in comparison with the compound alone. However, DCFH‐DA staining experiments indicated that no significant ROS was produced inside the cells. Consequently, photocytotoxicity investigations did not show any significant effect. Focus of future work will be the successful development of a nanobody‐containing Ru^II^ polypyridine conjugate with ROS and photocytotoxicity inside cancerous cells.

## Experimental Section

**Materials**: All chemicals were obtained from commercial sources and were used without further purification. Solvents were dried over molecular sieves if necessary. The Ru^II^ complexes [Ru(phen)_2_(dppz‐7‐aminomethyl)](PF_6_)_2_ 4a and [Ru(bipy)_2_(dppz‐OMe)](PF_6_)_2_ 2c were synthesized as previously reported.

**Instrumentation and methods**: ^1^H and ^13^C NMR spectra were recorded on a Bruker 400 MHz NMR spectrometer. ESI‐MS experiments were carried out using a LTQ‐Orbitrap XL from Thermo Scientific (Thermo Fisher Scientific, Courtaboeuf, France) and operated in positive ionization mode, with a spray voltage at 3.6 kV. No sheath and auxiliary gas was used. Applied voltages were 40 and 100 V for the ion transfer capillary and the tube lens, respectively. The ion transfer capillary was held at 275 °C. Detection was achieved in the Orbitrap with a resolution set to 100 000 (at *m*/*z* 400) and a *m*/*z* range between 150–2000 in profile mode. Spectrum was analyzed using the acquisition software XCalibur 2.1 (Thermo Fisher Scientific). The automatic gain control (AGC) allowed accumulation of up to 2×10^5^ ions for FTMS scans, Maximum injection time was set to 300 ms and 1 μscan was acquired. 10 μL was injected using a Thermo Finnigan Surveyor HPLC system (Thermo Fisher Scientific) with a continuous infusion of methanol at 100 μL min^−1^. For analytic and preparative HPLC the following system has been used: 2× Agilent G1361 1260 Prep Pump system with Agilent G7115A 1260 DAD WR Detector equipped with an Agilent Pursuit XRs 5C18 (Analytic: 100 Å, C18 5 μm 250 × 4.6 mm, Preparative: 100 Å, C_18_ 5 μm 250 ×300 mm) Column and an Agilent G1364B 1260‐FC fraction collector. The solvents (HPLC grade) were Millipore water (0.1 % TFA, solvent A) and acetonitrile (0.1 % TFA, solvent B). The sample was dissolved in 1:1 (*v*/*v*) CH_3_CN/ H_2_O 0.1 % TFA solution and filtered through a 0.2 μm membrane filter. Gradient: 0–3 min: isocratic 95 % A (5 % B); 3–17 min: linear gradient from 95 % A (5 % B) to 0 % A (100 % B); 17–25 min: isocratic 0 % A (100 % B). The flow rate was 1 mL min^−1^ (for preparative purposes: 20 mL min^−1^) and the chromatogram was detected at 250, 350, 450 nm.

**Synthesis**

**[Ru(phen)_2_(dppz‐7‐maleimidemethyl)](PF_6_)_2_**: The synthesis of [Ru(phen)_2_‐dppz‐7‐maleimidemethyl]^2+^ is already published4a but, in this study, a slightly different synthetic route was employed. [Ru(phen)_2_(dppz‐7‐aminomethyl)](PF_6_)_2_ (25 mg, 1.0 equiv) and maleic anhydride (46 mg, 20.0 equiv) were suspended in acetic acid (10 mL) under a nitrogen atmosphere. The mixture was held at reflux for 10 h. The solution was then cooled, and a saturated aqueous solution of NH_4_PF_6_ was added. The crude product, which precipitated as a PF_6_ salt, was collected by filtration and washed three times with H_2_O and Et_2_O. The product was purified by column chromatography on silica gel with a CH_3_CN/aq. KNO_3_ (0.4 m) solution (10:1). The fractions containing the product were united and the solvent was removed. The residue was dissolved in CH_3_CN and undissolved KNO_3_ was removed by filtration. The solvent was removed and the product was dissolved in H_2_O. Upon addition of NH_4_PF_6_ the product precipitated as a PF_6_ salt. The solid was obtained by centrifugation and was washed with H_2_O and Et_2_O. Yield: 86 %. Experimental data fits with the literature. Purity of the sample was assessed by NMR and HPLC analysis. RP‐HPLC: *t*_R_=16.2 min.

**[Ru(phen)_2_(dppz‐7‐maleimidemethyl‐S‐Cys‐(Ser)_2_(Gly)_5_‐NH_3_)](TFA)_3_**: [Ru(phen)_2_(dppz‐7‐maleimidemethyl)](PF_6_)_2_ (16 mg, 1.0 equiv) and (NH_2_CO‐Cys‐(Ser)_2_(Gly)_5_‐NH_3_)(TFA) (11.6 mg, 1.2 equiv) were dissolved in a 1:1 CH_3_CN/H_2_O mixture (20 mL) and stirred in the dark at room temperature. The progress of the reaction was followed via HPLC. After 24 h, additional (NH_2_CO‐Cys‐(Ser)_2_(Gly)_5_‐NH_3_)(TFA) (4.8 mg, 0.5 equiv) were added. The reaction mixture was stirred for another 6 h until the complete consumption of the Ru^II^ complex was monitored. The solvent was removed under reduced pressure and the product was purified by preparative HPLC. The product was isolated as a red TFA salt. Purity of the sample was assessed by HPLC analysis. Yield: 95 %. HRMS (ESI) *m*/*z*: calcd for [C_66_H_62_N_18_O_12_RuS‐3TFA]^3+^: 445.7874; found: 445.7875; RP‐HPLC: *t*_R_=14.9 min.

***Escherichia coli*** **strains and plasmids**: *E. coli* NEB 5‐alpha (*fhuA2* Δ(*argF‐lacZ*)*U169 phoA glnV44* Φ*80*Δ (*lacZ*)*M15 gyrA96 recA1 relA1 endA1 thi‐1 hsdR17*) was used in molecular cloning experiments, whereas *E. coli* SHuffle T7 Express (*fhuA2 lacZ::T7 gene1* [lon] *ompT ahpC gal* λ*att*::pNEB3‐r1‐*cDsbC* (Spec^R^, *lacI* ^*q*^) Δ*trxB sulA11 R(mcr‐73::miniTn10*–Tet^S^)2 [dcm] *R(zgb‐210::Tn10*–Tet^S^) *endA1* Δ*gor* Δ(*mcrC‐mrr*)*114::IS10*) and *E. coli* BL21(DE3) (*fhuA2* [lon] *ompT gal* (λ *DE3*) [dcm] Δ*hsdS*) were used for expression of the recombinant proteins. All strains were purchase from New England Biolabs. The generation of pET‐28b:7C12 encoding the EGFR‐specific single‐domain antibody 7C12 has been previously described.^31^ The plasmid pGBMCS‐SortA was a gift from Fuyuhiko Inagaki (Addgene plasmid #21931).^32^

**Molecular cloning**: A DNA fragment coding for a (GGGGS)_3_ spacer followed by a Strep‐tag, the LPETGG sortase motif and another (GGGGS)_3_ spacer was commercially synthesized including a 5′ restriction site for HindIII and a 3′ restriction site for XhoI, respectively. The ≈150‐nt fragment was digested with appropriate restriction endonucleases and ligated in‐frame into HindIII/XhoI‐linearized pET‐28b:7C12 plasmid.^31^ The ligation reactions were transformed into chemically competent *E. coli* NEB 5‐alpha cells. The DNA sequences of the resulting recombinant construct pET‐28b:7C12‐Strep‐Sortag‐His_6_ were checked by Sanger sequencing.

**Cultivation and expression of recombinant proteins**: Freshly transformed *E. coli* SHuffle T7 Express or *E. coli* BL21(DE3) harboring the plasmids pET‐28b:7C12‐Strep‐Sortag‐His_6_ or pGBMCS‐SortA were inoculated in 10 mL of LB broth containing 50 μg mL^−1^ of kanamycin or 100 μg mL^−1^ of ampicillin, respectively, and cultivated at 30 °C overnight in an orbital shaker with 50 mm offset and shaking speed of 200 rpm. After that, 5 mL of this pre‐culture were transferred into 125 mL MagicMedia™ *E. coli* Expression Medium (Life Technologies) in 1000 mL baffled‐bottom glass flasks and grown at 30 °C for 24 h. For final harvest, cultures were chilled on ice for 5 min and centrifuged for at least 15 min at 6000 *g* and 4 °C. After removal of the supernatant, cell pellets were either stored at −20 °C or subjected to purification procedure immediately.

**Purification of recombinant proteins**: A high‐capacity Ni‐iminodiacetic acid (IDA) resin in combination with an ÄKTA pure chromatography system (GE Healthcare) was used for purification of hexahistidine tagged proteins by immobilized metal affinity chromatography (IMAC) under native conditions. Efficient cell lysis was achieved by addition of 1 mL RIPA cell lysis buffer (G‐Biosciences) supplemented with EDTA‐free protease inhibitor cocktail (Roche Diagnostics), 500 μg lysozyme (Sigma–Aldrich) and 25 U endonuclease (Thermo Scientific Pierce) per 200 mg bacterial cell pellet. Prior to incubation on ice for at least 15 min, the pelleted cells were resuspended completely by vortexing or pipetting up and down until no cell clumps remained. After centrifugation at 10 000 *g* and 4 °C for 20 min to remove cellular debris, the clarified supernatant was loaded using an automated sample pump with a flow rate of 0.5 mL min^−1^. IMAC was performed on a prefilled 5‐mL His60 Ni Superflow cartridge (Clontech Laboratories) at a flow rate of 5 mL min^−1^ in equilibration buffer (50 mm Tris**⋅**HCl, 150 mm NaCl, pH 7.5). Before elution of the hexahistidine‐tagged proteins by addition of 8 CV elution buffer (50 mm Tris**⋅**HCl, 150 mm NaCl, 500 mm imidazole, pH 7.5), the column was washed with 8 CV equilibration buffer and 7 CV wash buffer (50 mm Tris**⋅**HCl, 150 mm NaCl, 35 mm imidazole, pH 7.5). Removal of imidazole and buffer exchange after IMAC was achieved by dialysis against sortase buffer (50 mm Tris**⋅**HCl, 150 mm NaCl and 10 mm CaCl_2_, pH 7.5) using a cellulose ester membrane with a molecular weight cut‐off of 3.5–5 kDa (Spectrum Laboratories).

**Gel electrophoresis**: Denaturing sodium dodecyl sulfate‐polyacrylamide gel electrophoresis (SDS‐PAGE) was carried out according to a standard protocol.^33^ For each gel, PageRuler Plus Prestained Protein Ladder (Thermo Fisher Scientific) was used as molecular weight ladder standard. After electrophoresis, gels were imaged with a D‐DiGit Gel Scanner (LI‐COR Biosciences) and subsequently stained with PageBlue protein staining solution (Thermo Fisher Scientific) according to the manufacturer's instructions.

**Protein determination**: Protein concentration was determined with the DC Protein Assay (Bio‐Rad Laboratories) according to the manufacture's microplate assay protocol using bovine serum albumin in sortase buffer (50 mm Tris**⋅**HCl, 150 mm NaCl and 10 mm CaCl_2_, pH 7.5) as protein standard.

**Sortase A‐mediated conjugation**: Small‐scale reactions were set up in 100 μL with variable molar ratios of SrtA, 7C12‐Strep‐Sortag‐His_6_ and [Ru(phen)_2_(dppz‐7‐maleimidemethyl‐S‐Cys‐(Ser)_2_(Gly)_5_‐NH_3_)]^3+^and different incubation times. The optimal conditions were upscaled and the reaction mixture was composed of 2 μmol SrtA, 2 μmol NB and 20 μmol [Ru(phen)_2_(dppz‐7‐maleimidemethyl‐*S*‐Cys‐(Ser)_2_(Gly)_5_‐NH_3_)]^3+^ in sortase buffer (50 mm Tris**⋅**HCl, 150 mm NaCl and 10 mm CaCl_2_, pH 7.5). Bioconjugation reactions were incubated at 30 °C for up to 6 h in the dark with gentle shaking.

**Purification of conjugation reactions**: In the first purification step, all remaining hexahistidine tagged proteins were eliminated from the reaction mixture by IMAC using prepacked His60 Ni Gravity Columns (Clontech Laboratories). After collection of the flow‐through, the gravity‐flow column was washed twice with equilibration buffer (50 mm Tris**⋅**HCl, 150 mm NaCl, pH 7.5). These wash fractions as well as the flow‐through were analyzed for the presence of the Ru‐NB conjugate by SDS‐PAGE. Remaining unconjugated [Ru(phen)_2_(dppz‐7‐maleimidemethyl‐S‐Cys‐(Ser)_2_(Gly)_5_‐NH_3_)]^3+^ was removed in a second purification step by size‐exclusion chromatography using Zeba Spin Desalting Columns (7 K MWCO, Thermo Scientific) with elution in PBS. The purified conjugate was sterile filtered using Whatman Puradisc FP 30 cellulose acetate syringe filter units with a pore size of 0.2 μm (GE Healthcare Life Sciences) and stored at 4 °C.

**Matrix‐assisted laser desorption ionization time‐of‐flight (MALDI‐TOF) mass spectrometry of purified sdAb conjugates**: 2,5‐Dihydroxyactetophenone (2,5‐DHAP, Bruker Daltonik) was used as matrix for MALDI‐TOF MS. For solubilization of the matrix, 7.6 mg of 2,5‐DHAP were dissolved in 375 μL of absolute ethanol. After this, 125 μL of an 18 mg mL^−1^ aqueous solution of diammonium hydrogen citrate (Sigma–Aldrich) were added. Protein samples were desalted using mixed cellulose esters membrane filters with a pore size of 0.025 μm and a diameter of 25 mm (MF‐Millipore Membrane Filter VSWP, Merck Chemicals). Briefly, the filter was placed on the water surface of a beaker filled with distilled water. A 2 μL aliquot of the protein sample was carefully pipetted on top of the membrane. After incubation at room temperature for at least 10 min, 2 μL of the dialyzed sample was mixed with 2 μL of 2 % TFA solution. After addition of 2 μL of the matrix solution, the mixture was pipetted up and down until the crystallization starts and the solution became cloudy. Finally, 0.5 μL of the crystal suspension was spotted onto the ground steel target plate and the droplet was air‐dried completely at room temperature.

Spectra were acquired with an autoflex II TOF/TOF (Bruker Daltonik) in positive linear mode in combination with the flexControl software (Version 3.3, Bruker Daltonik) and analyzed with the flexAnalysis software (Version 3.3, Bruker Daltonik). Theoretical molecular weights were calculated using the Compute pI/Mw tool on the ExPASy Server.^34^

**Spectroscopic measurements**: The absorption of the samples was measured in a cuvette with a Lambda 800 UV/VIS Spectrometer (PerkinElmer Instruments) or in 96 well plates with a SpectraMax M2 Spectrometer (Molecular Devices). The emission was measured by irradiation of the sample in fluorescence quartz cuvettes (width 1 cm) using a NT342B Nd‐YAG pumped optical parametric oscillator (Ekspla) at 450 nm. The luminescence was focused and collected at a right angle to the excitation pathway and directed to a Princeton Instruments Acton SP‐2300i monochromator. As a detector a XPI‐Max 4 CCD camera (Princeton Instruments) was used.

**Luminescence quantum yield measurements**: For the determination of the luminescence quantum yield, the samples were prepared in a CH_3_CN solution with an absorbance of 0.1 at 450 nm. This solution was irradiated in fluorescence quartz cuvettes (width 1 cm) using a NT342B OPO pulse laser Nd‐YAG pumped optical parametric oscillator (Ekspla) at 450 nm. The emission signal was focused and collected at a right angle to the excitation pathway and directed to a Princeton Instruments Acton SP‐2300i monochromator. As a detector a XPI‐Max 4 CCD camera (Princeton Instruments) was used. The luminescence quantum yields were determined by comparison with the reference [Ru(bipy)_3_]Cl_2_ in CH_3_CN (*Φ*_em_=0.059)^24^ applying the following formula [Eq. (1)]:

(1) $$\Phi {}_{\mathrm{em},\mathrm{sample}}=\Phi {}_{\mathrm{em},\mathrm{reference}}\times \frac{F{}_{\mathrm{reference}}}{F{}_{\mathrm{sample}}}\times \frac{I{}_{\mathrm{sample}}}{I{}_{\mathrm{reference}}}\times {\left(\frac{n{}_{\mathrm{sample}}}{n{}_{\mathrm{reference}}}\right)}^{2}F=1-10{}^{-A}$$

*Φ*_em_=luminescence quantum yield, *F*=fraction of light absorbed, *I*=integrated emission intensities, *n*=refractive index, *A*=absorbance of the sample at irradiation wavelength.

**Lifetime measurements**: For the determination of the lifetimes, the samples were prepared in an air saturated and in a degassed CH_3_CN solution with an absorbance of 0.1 at 450 nm. This solution was irradiated in fluorescence quartz cuvettes (width 1 cm) using a NT342B Nd‐YAG pumped optical parametric oscillator (Ekspla) at 450 nm. The emission signal was focused and collected at a right angle to the excitation pathway and directed to a Princeton Instruments Acton SP‐2300i monochromator. As a detector a R928 photomultiplier tube (Hamamatsu) was used.

**Singlet oxygen measurements**

**Direct evaluation**: The samples were prepared in an air‐saturated DMSO or D_2_O solution with an absorbance of 0.2 at 450 nm. This solution was irradiated in fluorescence quartz cuvettes (width 1 cm) using a mounted M450LP1 LED (Thorlabs) whose irradiation, centered at 450 nm, was focused with aspheric condenser lenses. The intensity of the irradiation was varied using a T‐Cube LED Driver (Thorlabs) and measured with an optical power and energy meter. The emission signal was focused and collected at a right angle to the excitation pathway and directed to a Princeton Instruments Acton SP‐2300i monochromator. A long‐pass glass filter was placed in front of the monochromator entrance slit to cut off light at wavelengths shorter than 850 nm. The slits for detection were fully open. As a detector an EO‐817 L IR‐sensitive liquid nitrogen cooled germanium diode detector (North Coast Scientific Corp.) was used. The singlet oxygen luminescence at 1270 nm was measured by recording spectra from 1100 to 1400 nm. For the data analysis, the singlet oxygen luminescence peaks at different irradiation intensities were integrated. The resulting areas were plotted against the percentage of the irradiation intensity and the slope of the linear regression calculated. The absorbance of the sample was corrected with an absorbance correction factor. As reference for the measurement in an CH_3_CN solution phenalenone (*Φ*_phenaleone_=0.95)^35^ and for the measurement in a D_2_O solution [Ru(bipy)_3_]Cl_2_ (*Φ* ${}_{\mathrm{Ru}\left(\mathrm{bipy}\right){}_{3}\mathrm{Cl}{}_{2}}$ =0.22)^36^ was used and the singlet oxygen quantum yields were calculated using the following formula [Eq. (2)]:

(2) $$\Phi {}_{\mathrm{sample}}=\Phi {}_{\mathrm{reference}}\times \frac{S{}_{\mathrm{sample}}}{S{}_{\mathrm{reference}}}\times \frac{I{}_{\mathrm{reference}}}{I{}_{\mathrm{sample}}}I=I{}_{0}\times \left(1-10{}^{-A}\right)$$

*Φ*=singlet oxygen quantum yield, *S*=slope of the linear regression of the plot of the areas of the singlet oxygen luminescence peaks against the irradiation intensity, *I*=absorbance correction factor, *I*_0_=light intensity of the irradiation source, *A*=absorbance of the sample at irradiation wavelength.

**Indirect evaluation**: For the measurement in DMSO: The samples were prepared in an air‐saturated DMSO solution containing the complex with an absorbance of 0.2 at the irradiation wavelength and 1,3‐diphenylisobenzofuran (DPBF, 30 μm). For the measurement in PBS buffer: The samples were prepared in an air‐saturated PBS solution containing the complex with an absorbance of 0.2 at the irradiation wavelength, *N*,*N*‐dimethyl‐4‐nitrosoaniline aniline (RNO, 20 μm) and histidine (10 mm). The samples were irradiated on 96 well plates with an Atlas Photonics LUMOS BIO irradiator for different times. The absorbance of the samples was measured during these time intervals with a SpectraMax M2 Microplate Reader (Molecular Devices). The difference in absorbance (*A*_0_−*A*) at 415 nm for the DMSO solution and at 440 nm for the PBS solution was measured and plotted against the irradiation times. From the plot the slope of the linear regression was calculated as well as the absorbance correction factor determined. The singlet oxygen quantum yields were calculated using the same formulas as used for the direct evaluation.

**Cell culture**: Cell culture flasks, dishes and plates (CELLSTARS) were supplied by Greiner Bio‐One GmbH. The adherent human tumor cell lines A431 (ATCC number: CRL‐1555) and MDA‐MB 435S (ATCC number: HTB‐129) were maintained as previously reported.^31^, ^37^ All cell lines were confirmed to be mycoplasma‐negative using the Venor GeM Advance Mycoplasma Detection Kit (Minerva Biolabs) and were tested monthly.

**Cell uptake studies**: A total of 300 000 MDA‐MB 435S cells and 450 000 A431 cells were seeded in T25 cell culture flasks in 5 mL DMEM supplemented with 10 % fetal calf serum (FCS), respectively, and incubated in a humidified atmosphere of 95 % air/5 % CO_2_ at 37 °C. After 48 h of incubation, cells were washed twice with warm PBS. The buffer was then replaced by fresh DMEM supplemented with 10 % FCS and different concentrations of the Ru‐NB conjugate or [Ru(bipy)_2_(DPPZ‐OMe)](PF_6_)_2_. Following incubation at 37 °C for certain time periods, medium was removed and the cells washed three times with warm PBS and trypsinized. After resuspension in warm DMEM with 10 % FCS, the pellets were collected by centrifugation at 200 *g* for 5 min and washed once with warm PBS. The cell pellets were resuspended in 500 μL of PBS, lysed by ten freeze‐thaw cycles, and sonicated in an ice‐cold ultrasonic bath for 20 min (SONOREX SUPER 10P digital, Bandelin). After determination of the protein content, the lysates were lyophilized on an Alpha 2–4 LSC plus (CHRIST).

**ICP‐MS studies**: After digestion of samples in distilled ultrapure 65 % HNO_3_ (Roth) and dilution in 1 % HNO_3_, ICP‐MS measurements were performed on an iCap RQ ICP‐MS spectrometer (Thermo Fisher Scientific) equipped with a SC‐2DX autosampler (ESI). Calibration was done with Ru single element standard (Merck 170347). Rh and Sc were used as internal standards. Limit of detection (LOD) was 50 ng L^−1^ Ru.

**Confocal microscopy**: A total of 100 000 A431 cells were seeded in 35 mm imaging dishes (IBIDI) in 2 mL DMEM supplemented with 10 % fetal calf serum (FCS), and incubated in a humidified atmosphere of 95 % air/5 % CO_2_ at 37 °C. After 24 h of incubation, media was refreshed and cells were incubated with 100 nm of Ru‐NB at 37 °C for up to 48 h. Afterward, cells were washed thrice with ice‐cold PBS, fixed with 4 % paraformaldehyde and 2.5 % sucrose in PBS, and permeabilized with 0.25 % TritonX‐100 in PBS for 10 min. To prevent unspecific antibody binding, cells were incubated with 10 % FCS in PBS overnight at 4 °C. Cells were then incubated with rabbit anti‐EGFR (D38B1) Alexa Fluor 647 monoclonal antibody (Cell Signaling Technology) and with StrepMAB‐Classic Chromeo 488 conjugate (IBA Lifesciences) for 2 h at RT in the dark. Cells were again washed three times with PBS, and the nuclei were stained using Hoechst 33258. Fluorescence microscopy was performed with the Fluoview 1000 confocal laser scanning microscope (Olympus) using a 60× (NA 1.35) oil objective.

**Dark cytotoxicity and phototoxicity**: The dark and light cytotoxicity of the Ru^II^‐containing conjugates was assessed by fluorometric cell viability assay using resazurin (ACROS Organics). For dark and light cytotoxicity with the EGFR internalization step,^38^ A431 cells were seeded in triplicates in 96‐well plates at a density of 4000 cells per well in 100 μL, 24 h prior to treatment. Cells were then treated with serum free DMEM medium containing 0.3 % of BSA for 1 h at 37 °C. The medium was then replaced with increasing concentrations of Ru‐NB, then cells were incubated on ice for 1 h. After that time, cells were transferred for 1 h at 37 °C. The medium was then replaced by fresh complete medium. For the dark and light cytotoxicity without the EGFR internalization step, A431 cells were seed in triplicates in 96‐well plates at a density of 4000 cells per well in 100 μL, 24 h prior to treatment. The medium was then replaced with increasing concentrations of Ru‐NB for 44 h.

Cells used for the light cytotoxicity experiments with Ru‐NB were exposed to 480 nm light for 6×3.5 min with 15 min gap in between irradiations or in a 96‐well plate using a LUMOS‐BIO photoreactor (Atlas Photonics). Each well was individually illuminated with a 5 lm LED at constant current (6.741 J cm^−2^). After 44 h in the incubator, the medium was replaced by fresh complete medium containing resazurin (0.2 mg mL^−1^ final concentration). After 4 h incubation at 37 °C, the fluorescence signal of the resorufin product was read by SpectraMax M5 microplate reader (*λ*_ex_=540 nm, *λ*_em_=590 nm). IC_50_ values were calculated using GraphPad Prism software.

**Cellular ROS production**: 10 cm cell culture plates were seeded with A431 cell line and allowed to adhere overnight. Next, the cells were incubated with a DCFH‐DA solution (100 μm) in DMEM media for 30 min at 37 °C. Cells were then washed and treated with serum‐free DMEM medium containing 0.3 % of BSA for 1 h at 37 °C. The medium was then replaced in the plates with either Ru‐NB dilution, 0.1 mm H_2_O_2_ or medium. Cells were then incubated on ice for 1 h. After that time, the cells were transferred for 1 h at 37 °C. The medium was then replaced by fresh complete medium. The cells used for the light experiments were exposed to 480 nm light for 3.5 min using a LUMOS‐BIO photoreactor (Atlas Photonics; 1.124 J cm^−2^). All cells were then washed, collected and gated using Fortessa instrument in Cytometry Platform at the Curie Institute. Data were analyzed by using FlowJo 10.5.2 software.

## Conflict of interest

*The authors declare no conflict of interest*.

## Supporting information

As a service to our authors and readers, this journal provides supporting information supplied by the authors. Such materials are peer reviewed and may be re‐organized for online delivery, but are not copy‐edited or typeset. Technical support issues arising from supporting information (other than missing files) should be addressed to the authors.

Supplementary
