# Supplementary material for: Synthesis and Characterization of an Epidermal Growth Factor Receptor‐Selective RuII Polypyridyl–Nanobody Conjugate as a Photosensitizer for Photodynamic Therapy
Source: Chembiochem. 2019 Oct 22;21(4):531–42. doi: 10.1002/cbic.201900419 (PMC7065149; doi:10.1002/cbic.201900419)
Supplement: Supplementary file 1 — Supplementary [file CBIC-21-531-s001.pdf]

CHEMBIOCHEM

Supporting Information

Synthesis and Characterization of an Epidermal Growth Factor Receptor-Selective Ru^{II} Polypyridyl–Nanobody Conjugate as a Photosensitizer for Photodynamic Therapy

Johannes Karges+,[a] Marta Jakubaszek+,[a, b] Cristina Mari+,[c] Kristof Zarschler+,*[d]
Bruno Goud,[b] Holger Stephan,[d] and Gilles Gasser*[a]

cbic_201900419_sm_miscellaneous_information.pdf

Table of Content:

Figure S1. HPLC chromatogram of [Ru(phen)$_2$(dppz-7-maleimidemethyl)]$^{2+}$. S3

Figure S2. HPLC chromatogram of [Ru(phen)$_2$(dppz-7-maleimidemethyl-S-Cys-(Ser)$_2$(Gly)$_5$-NH$_3$)]$^{3+}$. S3

Figure S3. SDS-PAGE analysis of the reaction efficiency for chemoenzymatic conjugation of the [Ru(phen)$_2$(dppz-7-maleimidemethyl-S-Cys-(Ser)$_2$(Gly)$_5$-NH$_3$)]$^{3+}$ to the EGFR-specific NB 7C12. S4

Figure S4. SDS-PAGE analysis of the reaction efficiency for chemoenzymatic conjugation of the [Ru(phen)$_2$(dppz-7-maleimidemethyl-S-Cys-(Ser)$_2$(Gly)$_5$-NH$_3$)]$^{3+}$ to the EGFR-specific NB 7C12. S5

Figure S5. MALDI-TOF mass spectra of (A) purified NB-conjugate 7C12-Strep-[Ru(phen)$_2$(dppz-7-maleimidemethyl-S-Cys-(Ser)$_2$(Gly)$_5$-NH$_3$)]$^{3+}$ and (B) 7C12-Strep-Sortag-His$_6$. S6

Figure S6. Normalised UV/Vis spectra. S7

Figure S7. Emission spectra of **Ru-NB** in DMSO. S7

Figure S8. Lifetime spectra of **Ru-NB** in degassed DMSO. S8

Figure S9. Lifetime spectra of **Ru-NB** in aerated DMSO. S8

Figure S10. Amount of cell-associated ruthenium after incubation of A431 and MDA-MB-435S cells with 2 or 20 µM of Ru(bipy)$_2$(DPPZ-OMe) (PF$_6$)$_2$ for up to 48 h. S9

Figure S11. Cytotoxicity of **Ru-NB** in A431 cell line. S10

Figure S12. Cytotoxicity of **Ru-NB** in A431 cell line. S10

Figure S13. Cellular ROS production in A431 cells treated with **Ru-NB** and stained with DCFH-DA. S10

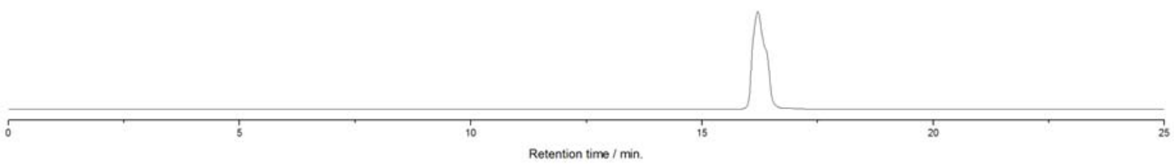

Figure S1. HPLC chromatogram of [Ru(phen)$_2$(dppz-7-maleimidemethyl)]$^{2+}$.

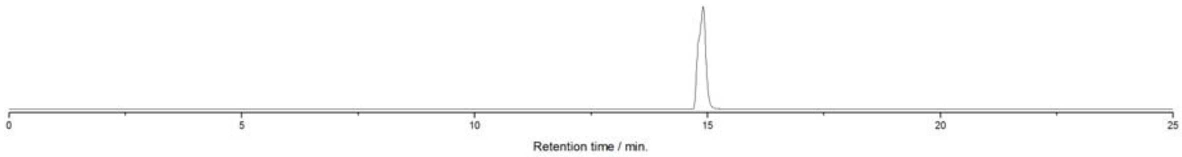

Figure S2. HPLC chromatogram of [Ru(phen)$_2$(dppz-7-maleimidemethyl-S-Cys-(Ser)$_2$(Gly)$_5$-NH$_3$)]$^{3+}$.

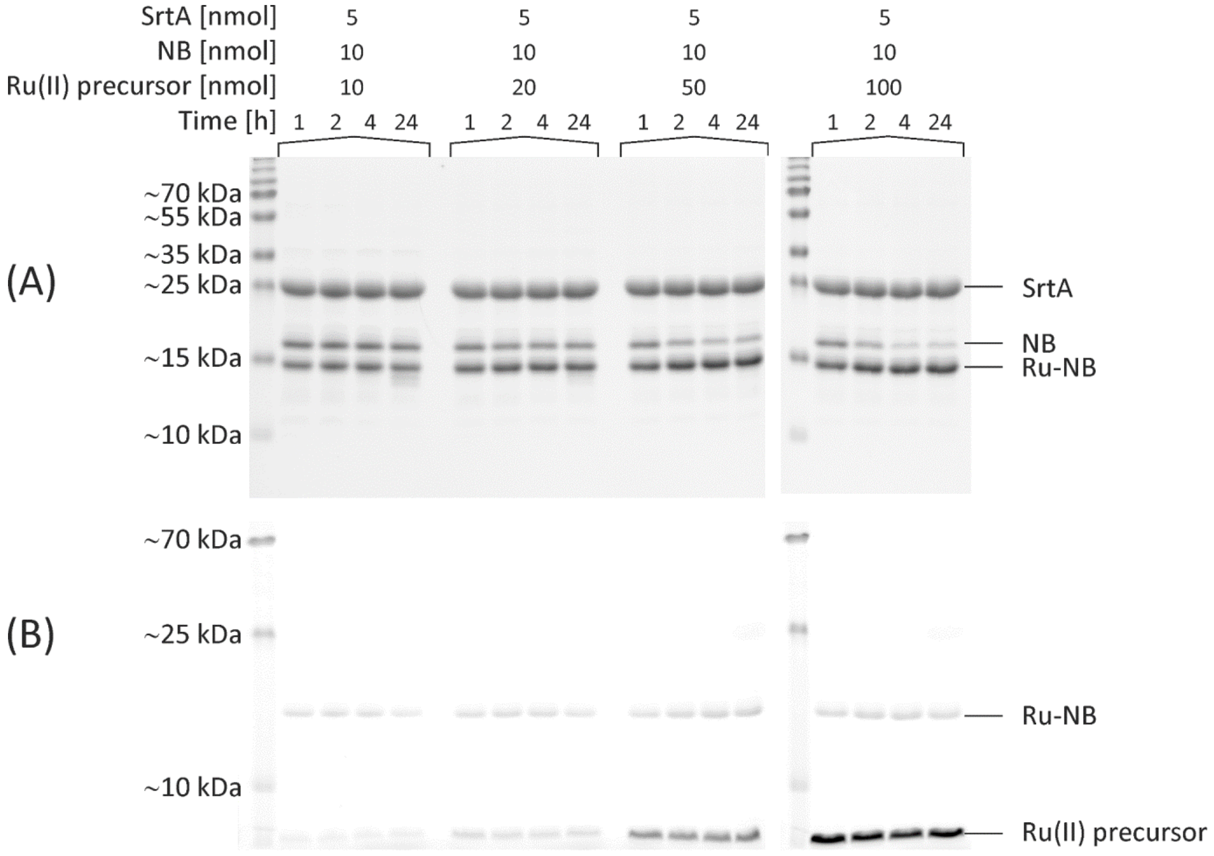

Figure S3. SDS-PAGE analysis of the reaction efficiency for chemoenzymatic conjugation of the $[Ru(phen)_2(dppz\text{-}7\text{-}maleimidemethyl\text{-}S\text{-}Cys\text{-}(Ser)_2(Gly)_5\text{-}NH_3)]^{3+}$ to the EGFR-specific NB 7C12. The amounts used were 5 nmol SrtA, 10 nmol NB and 10-100 nmol of Ru(II) precursor. The reaction was monitored for up to 24 h and aliquots were separated on 15% SDS polyacrylamide gels. After electrophoresis, gels were imaged with a D-DiGit Gel Scanner (B) to detect the signal of the Ru(II) complex and subsequently stained with colloidal Coomassie G-250 (A).

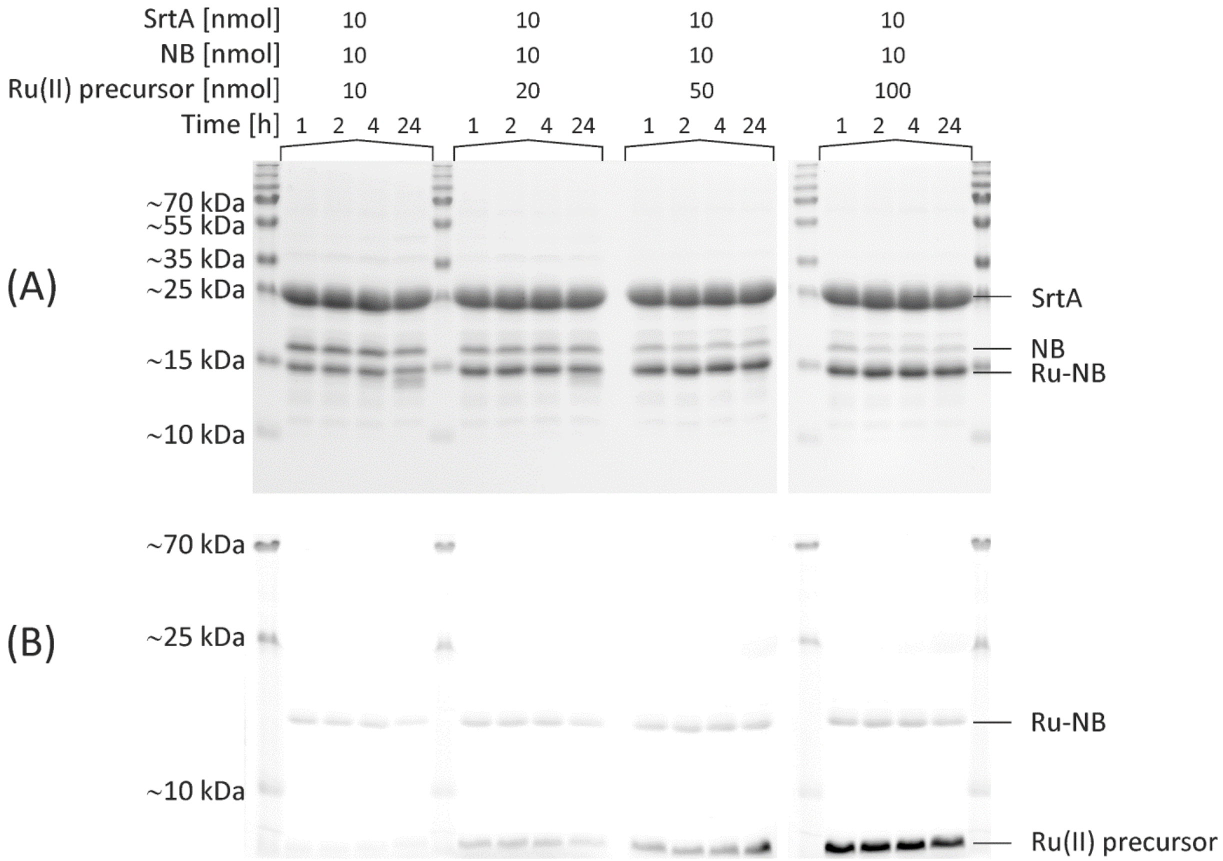

Figure S4. SDS-PAGE analysis of the reaction efficiency for chemoenzymatic conjugation of the $[Ru(phen)_2(dppz\text{-}7\text{-}maleimidemethyl\text{-}S\text{-}Cys\text{-}(Ser)_2(Gly)_5\text{-}NH_3)]^{3+}$ to the EGFR-specific NB 7C12. The amounts used were 10 nmol SrtA, 10 nmol NB and 10-100 nmol of Ru(II) precursor. The reaction was monitored for up to 24 h and aliquots were separated on 15% SDS polyacrylamide gels. After electrophoresis, gels were imaged with a D-DiGit Gel Scanner (B) to detect the signal of the Ru(II) complex and subsequently stained with colloidal Coomassie G-250 (A).

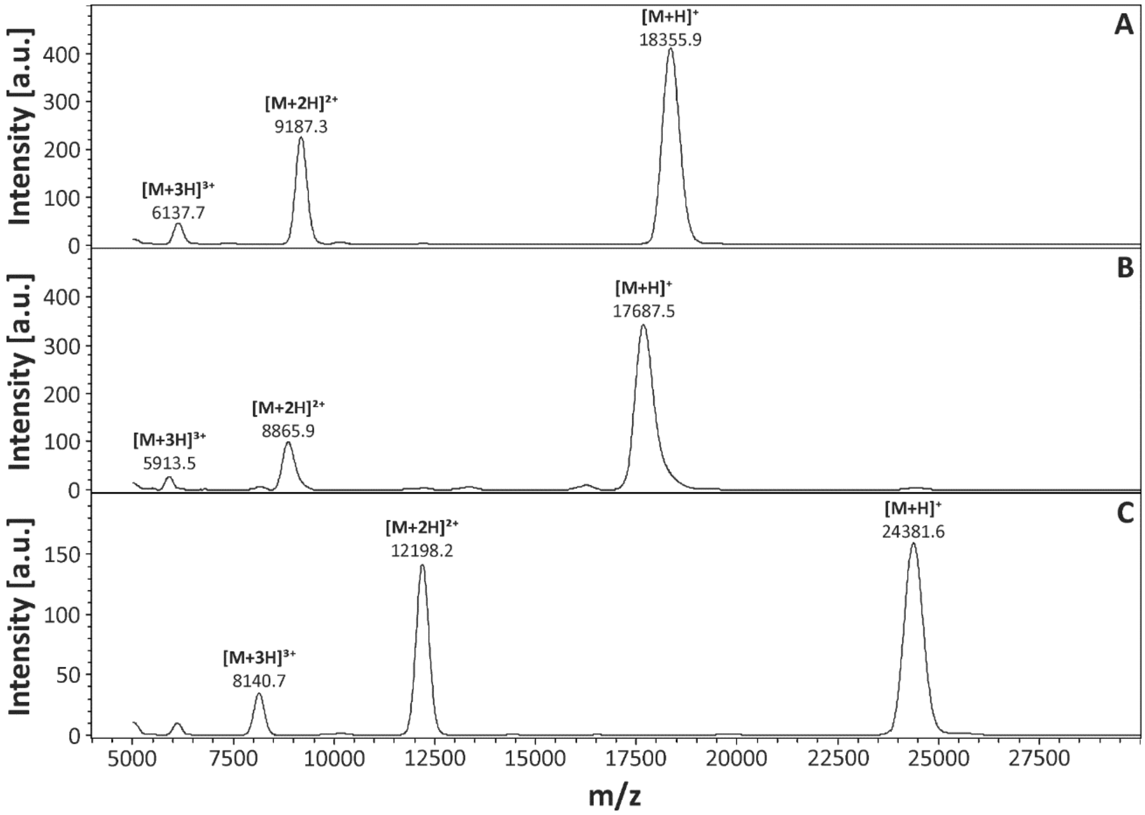

Figure S5. MALDI-TOF mass spectra of the purified (A) NB derivative 7C12-Strep-Sortag-His$_6$, (B) single-conjugated NB-conjugate 7C12-Strep-[Ru(phen)$_2$(dppz-7-maleimidemethyl-S-Cys-(Ser)$_2$(Gly)$_5$-NH$_3$)]$^{3+}$ (**Ru-NB**) and (C) sortase enzyme SrtA.

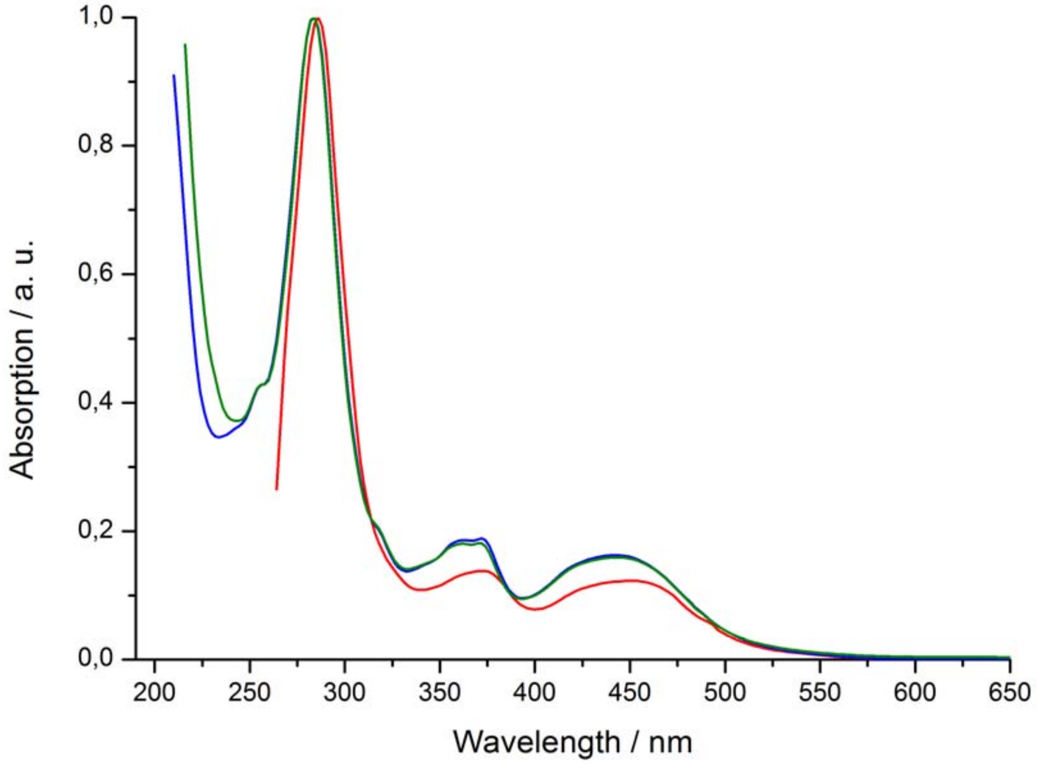

Figure S6. Normalised UV/Vis spectra of [Ru(phen)$_2$(dppz-7-maleimidemethyl)](PF$_6$)$_2$ in CH_3CN (blue), [Ru(phen)$_2$(dppz-7-maleimidemethyl-S-Cys-(Ser)$_2$(Gly)$_5$-NH$_3$)](TFA)$_3$ in CH_3CN (green) and **Ru-NB** in DMSO (red).

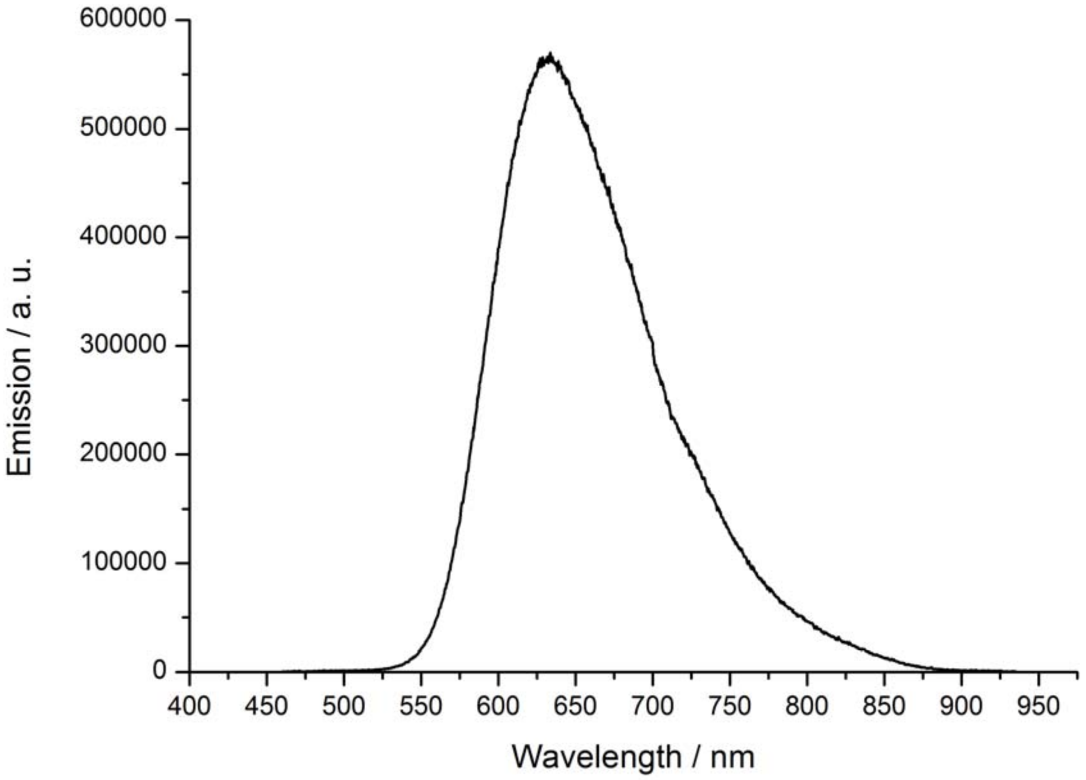

Figure S7. Emission spectra of **Ru-NB** in DMSO.

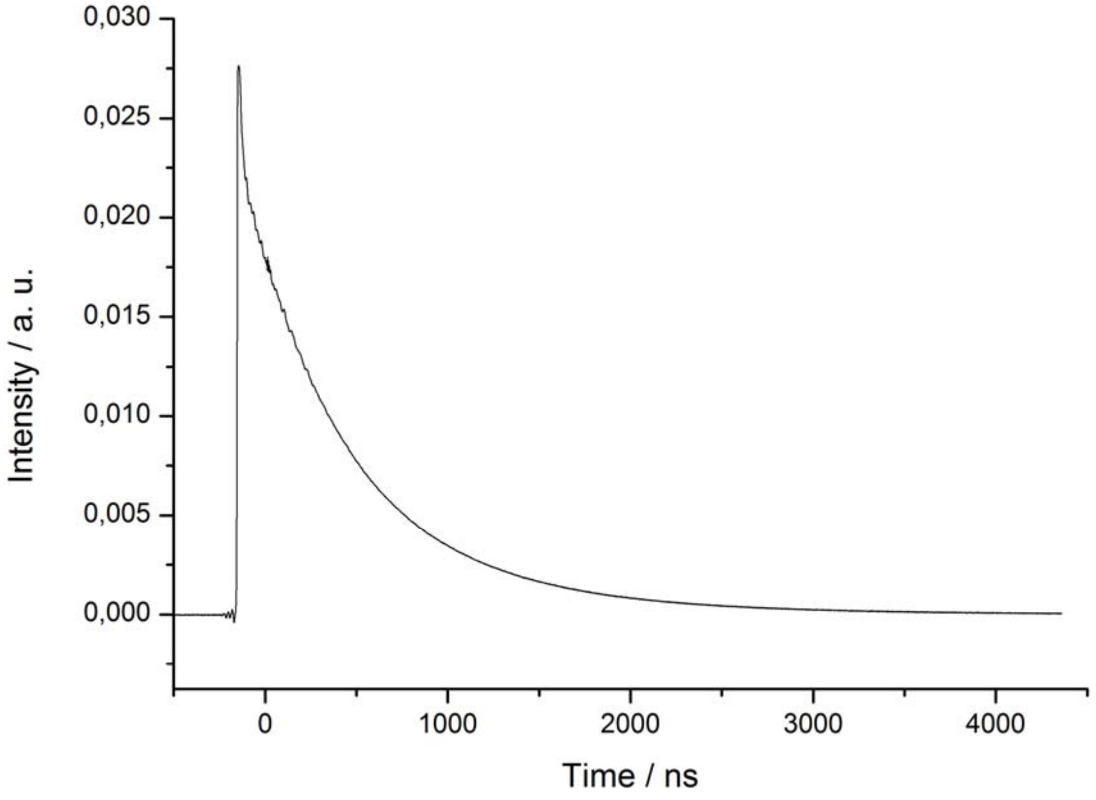

Figure S8. Lifetime spectra of **Ru-NB** in degassed DMSO.

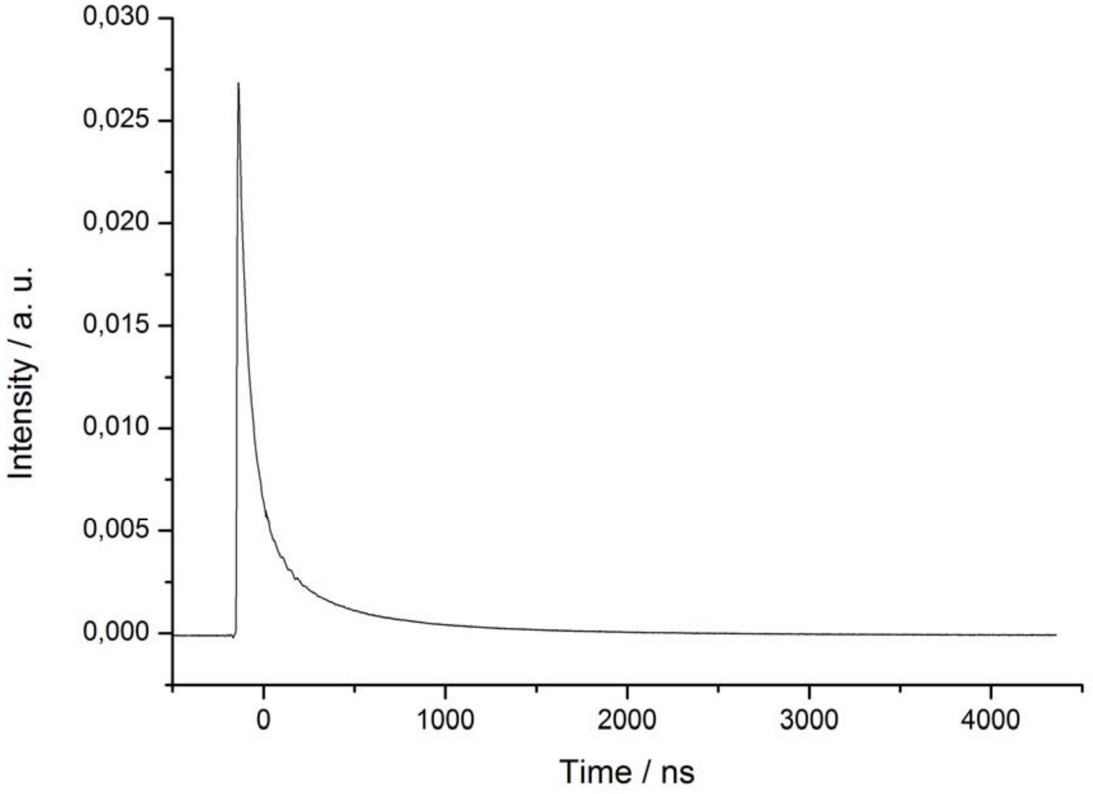

Figure S9. Lifetime spectra of **Ru-NB** in aerated DMSO.

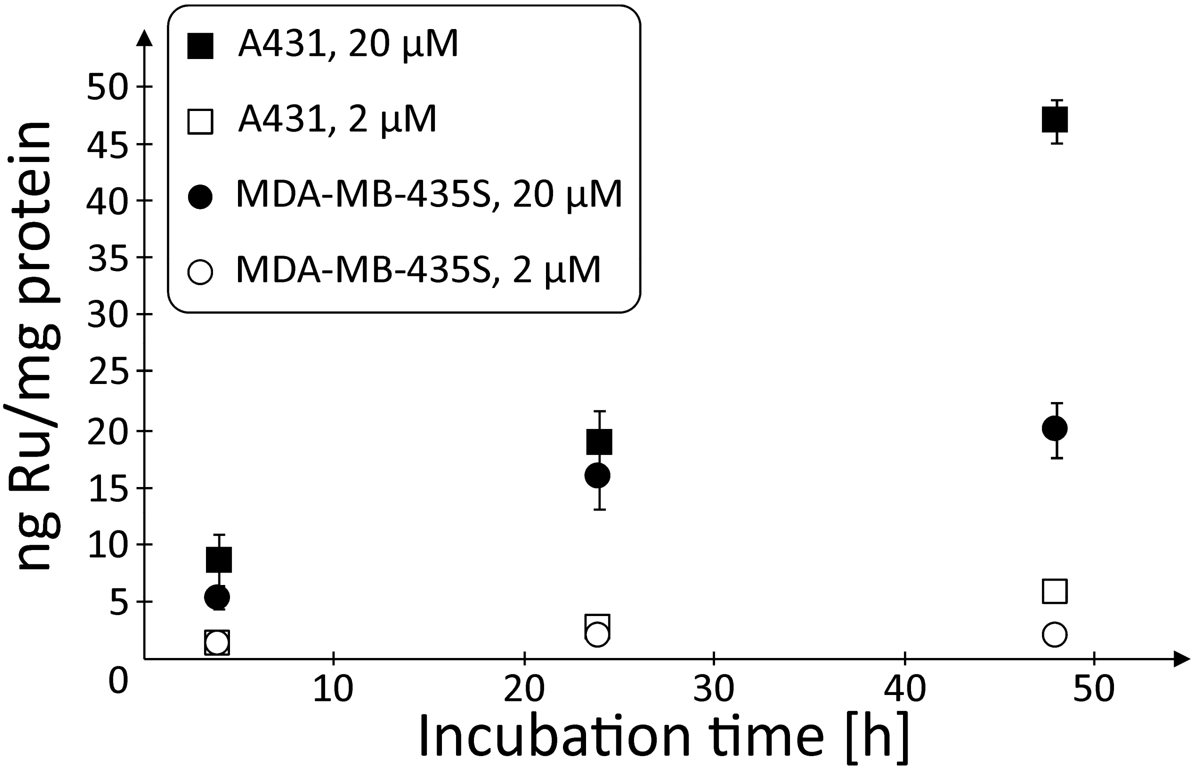

Figure S10. Amount of cell-associated ruthenium after incubation of A431 and MDA-MB-435S cells with 2 or 20 µM of Ru(bipy)$_2$(DPPZ-OMe) (PF$_6$)$_2$ for up to 48 h.

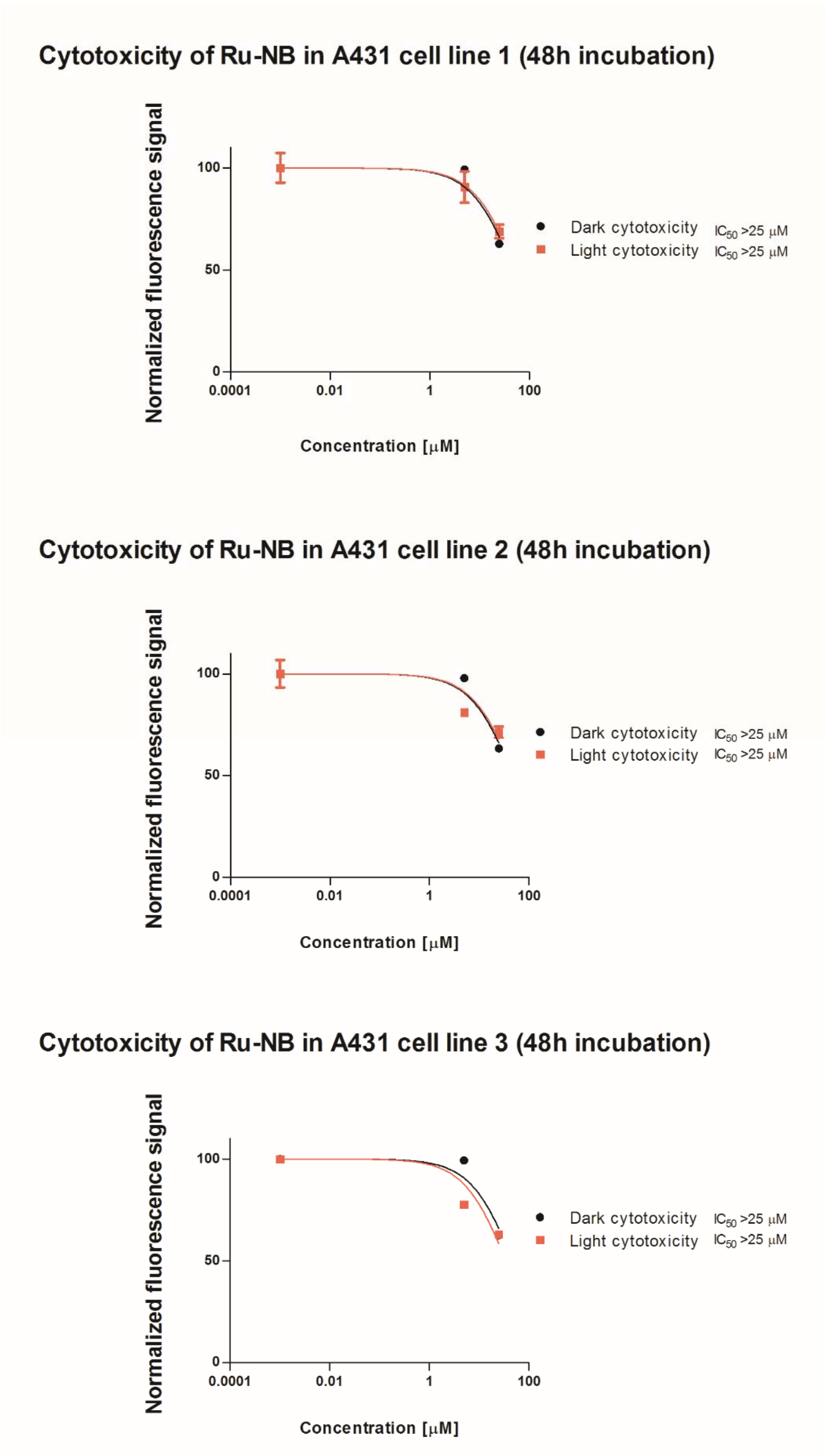

Figure S11. Cytotoxicity of **Ru-NB** in A431 cell line. Cells were treated for 48h, light irradiation: 6x 3.5 min at 480 nm.

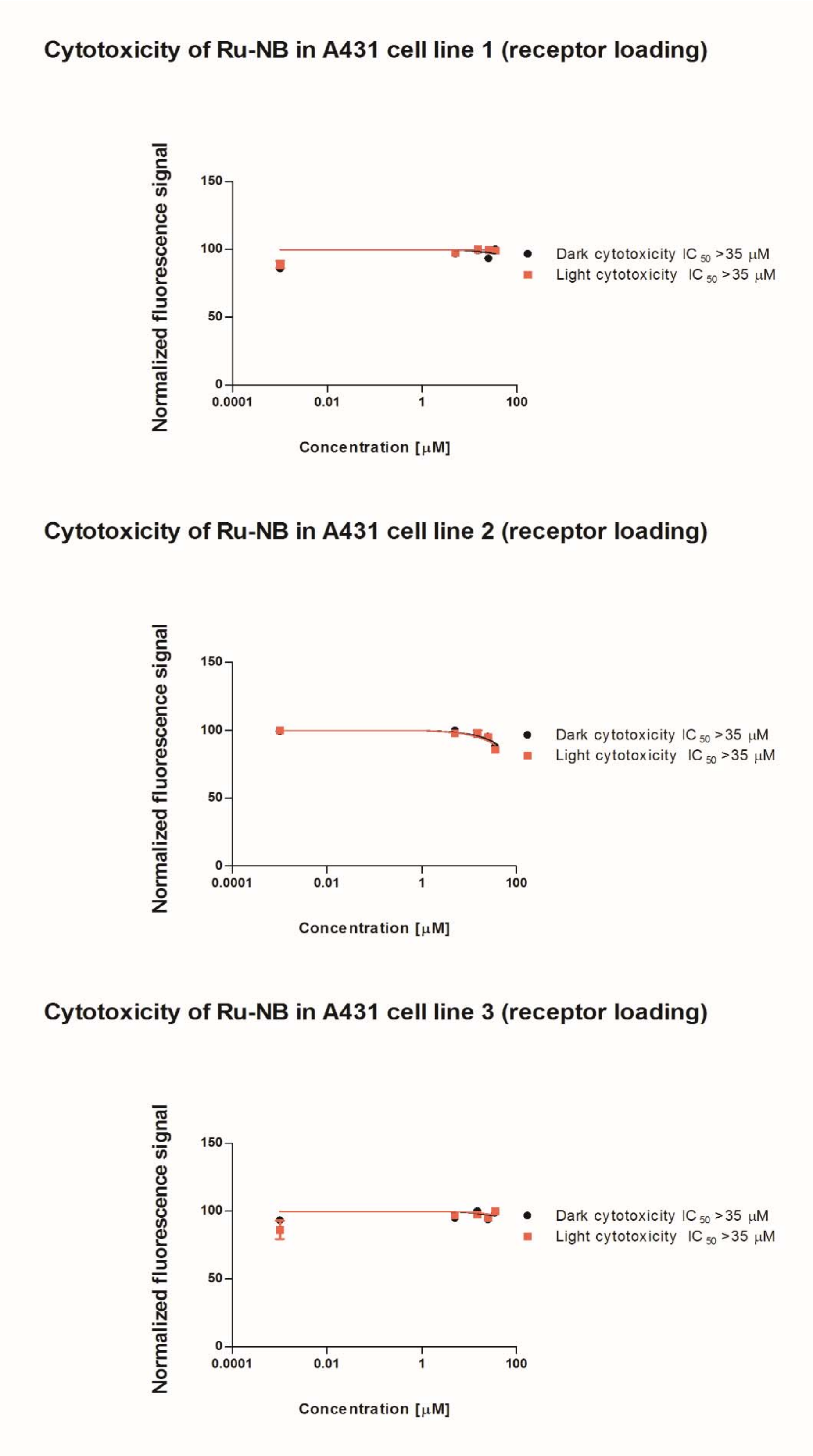

Figure S12. Cytotoxicity of **Ru-NB** in A431 cell line. Cells were treated using receptor internalisation protocol, light irradiation: 6x 3.5 min at 480 nm.

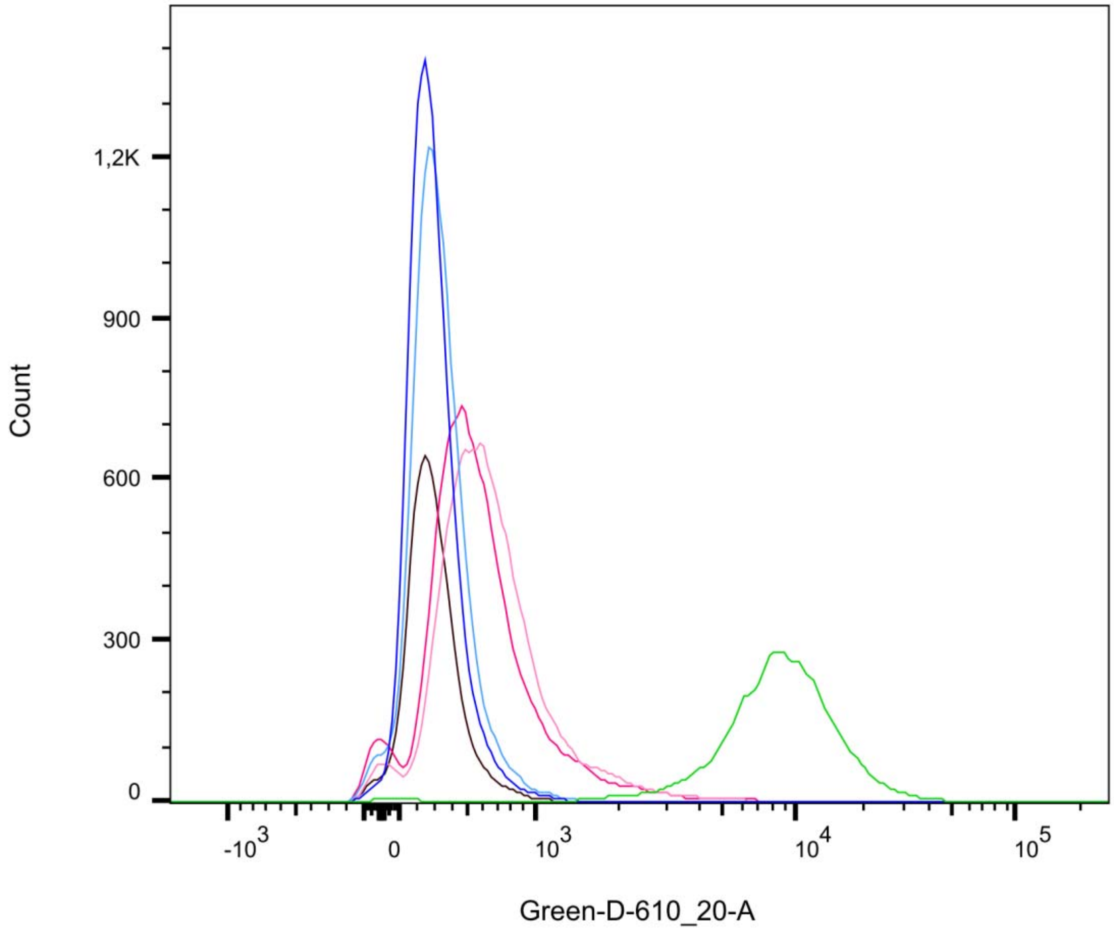

	Sample Name	Count	Mean : Green-D-610_20-A
■	DCFH-DA stained- H2O2 treated.fcs	10000	10186
■	DCFH-DA stained- irradiated.fcs	20000	319
■	DCFH-DA stained-not irradiated.fcs	20000	353
■	DCFH-DA stained- Ru(II) conjugate treated- not irradiated.fcs	20000	843
■	DCFH-DA stained- Ru(II) conjugate- irradiated.fcs	20000	753
■	Cells only.fcs	10000	317

Figure S13. Cellular ROS production in A431 cells treated with **Ru-NB and** stained with DCFH-DA. Cells were gated for DCFH-DA signal (Green-D-610_20-A) using flow cytometry. Cell count for each experimental group with mean of the DCFH-DA signal is provided in the table.
